# Transesophageal echocardiography during cardiopulmonary resuscitation is associated with alternate areas of compression: Analysis of healthcare provider experiences with potential implications for conventional compressions

**DOI:** 10.1371/journal.pone.0339974

**Published:** 2026-01-20

**Authors:** Rory A. Marshall, Kate DeVito-Porter, Geneviève Dallaire, Tom Jelic, Amanda Smith, Frank Myslik, Rohit Mohindra, Rajiv Thavanathan, Tracy D. Wilson, Paul Olszynski

**Affiliations:** 1 Physical Medicine and Rehabilitation, University of Michigan, Ann Arbor, Michigan, United States of America; 2 Department of Emergency Medicine, University of Saskatchewan, Saskatoon, Saskatchewan, Canada; 3 Medicine and Health Sciences Faculty, Université de Sherbrooke, Sherbrooke, Quebec, Canada; 4 Department of Emergency Medicine, Rady Faculty of Health Sciences, University of Manitoba, Winnipeg, Manitoba, Canada; 5 Department of Emergency Medicine, Schulich School of Medicine, London, Ontario, Canada; 6 North York General Hospital, Toronto, Ontario, Canada; 7 Schwartz Reisman Emergency Institute, Toronto, Ontario, Canada; 8 Division of Emergency Medicine, Department of Medicine, University of Toronto, Toronto, Ontario, Canada; 9 First60 FIRST60: Prehospital, Trauma and Resuscitation Sciences, Toronto, Ontario, Canada; 10 Department of Emergency Medicine, The Ottawa Hospital, Ottawa, Ontario, Canada; Medical University of Vienna, AUSTRIA

## Abstract

**Background:**

Ultrasound-guided cardiopulmonary resuscitation (UG-CPR) offers healthcare providers real-time, sonographic guidance during CPR. Transesophageal echocardiography-guided CPR (TEE-CPR) is limited to institutions with the prerequisite equipment and expertise. Learnings from TEE-CPR have the potential to improve conventional CPR methods, particularly in identification of alternate areas of compression (AOC) when conventional compressions fail. It is unclear how often, and to what extent, performing TEE-CPR compressions varies from conventional compressions. This study explored and compared healthcare provider impressions of performing chest compressions during conventional and TEE-CPR.

**Methods:**

An online survey was distributed to healthcare providers working at TEE-CPR sites throughout Canada. The 34-item survey explored commonalities and differences between conventional and TEE-CPR. Quantitative and qualitative analyses were used to describe changes in the AOC, compression dynamics including changes in chest wall and compression effort, and logistical differences associated with integration into broader resuscitative choreography.

**Results:**

Amongst 30 respondents from 5 distinct sites, 96.7% reported instances of TEE-CPR where the AOC was moved away from the conventional site to improve chest compression quality. Further, 76.7% of respondents indicated altering the AOC during at least half of TEE-CPR events. Alternate areas of compression were more common than the conventional AOC after initiation of TEE-CPR (pre-ultrasound conventional 25: alternative 5 versus post-ultrasound conventional 13: alternative 17, X2 (1,N = 30)=10.3, p = 0.0013). The reported shift was predominantly leftward (61.0%), then caudal (26.2%). Providers reported improved chest compression quality from real-time visual feedback. Most providers reported similar exertional effort while performing chest compressions.

**Conclusions:**

Use of TEE-CPR often leads to adjustments in chest compression location, predominantly in a leftward and/or caudal direction. Performing TEE-CPR compressions was reported to have minimal impact on provider exertion as compared to conventional CPR. Prospective research mapping the locations and frequencies of alternate AOCs during TEE-CPR, and associated clinical outcomes, are warranted.

## Introduction

Despite an emphasis on high quality cardiopulmonary resuscitation (CPR) [1,2], and improved availability of automated defibrillators [3], survival rates for out-of-hospital cardiac arrests (OHCA) remain low [4]. Despite the heterogeneity of OHCA patients [5], uniformly performed chest compressions and basic common interventions remain a cornerstone of CPR [6]. Current guidelines advise that CPR should be performed with the area of compression (AOC) over the lower half of the sternum (weak recommendation with very-low certainty evidence) [6,7].

It is theorized that a combination of intrathoracic pressure changes during the compression cycle (thoracic pump theory), and direct compression of the heart (cardiac pump theory) non-competitively generate forward blood flow during conventional CPR [8]. An emerging body of research suggests that the current AOC in conventional CPR often results in obstruction of key outflow structures [9], raising questions about the appropriateness of the current AOC [10–12].

Imaging research has demonstrated that the middle of the left ventricle often lies beyond the borders of lower half of the sternum [13–18]. Simultaneously, a growing body of preclinical work has reported that compressions directly over the left ventricle were associated with improved hemodynamics (end-tidal carbon dioxide, blood pressure, and cerebral blood velocity [19,20]). Compressions over the left ventricle may reduce outflow resistance, increase stroke volume, and cause transient compression of the descending aorta, indirectly apportioning more blood flow to the coronary and cranial circulation [19,20]. Mixed but promising findings have been mirrored in a few pilot clinical studies [21,22] indicating that targeting the left ventricle during CPR may already be showing clinical benefits. Ultrasound-guided cardiopulmonary resuscitation (UG-CPR) has been utilized to assess CPR efficacy during compressions [23–29]. Transesophageal Echocardiography guided CPR (TEE-CPR, one form of UG-CPR) further enables resuscitation teams to maximize compression of the left ventricle, identify and alleviate outflow obstruction, all while reducing duration of pulse checks [24–29].

While TEE-CPR enables real-time, continuous imaging to monitor and improve CPR efforts, the need for specialized equipment and training inhibits the use of TEE-CPR in the vast majority of settings worldwide (i.e., out-of-hospital, most in-hospital departments). This is further compounded by the fact that most OHCA patients die either on scene or in the emergency department [5,30], having received only conventional CPR over the conventional AOC. As paramedics are typically the first trained-healthcare professionals to deliver CPR for the majority of cardiac arrests [2,5,30], limiting CPR delivery to the conventional AOC may not be optimal for many patients. Learnings from TEE-CPR could help inform alternative areas and/or methods of compression.

Despite the clinical utility and increasing adoption of TEE-CPR, to date there has been minimal research detailing TEE-CPR alterations to chest compressions, specifically from the compressor’s perspective. This perspective is valuable as insights regarding changes to compression biomechanics may otherwise be missed, potentially losing translational learnings such as an alternate AOC for select patients. If improvements to chest compressions could be generalized from TEE-CPR data, such as the most common alternate AOC, then first-responder compressions could be improved for a large number of patients.

This survey was developed to gather insights into the specifics of TEE-CPR chest compressions with the outlook that learnings may spur on prospective data collection related to alterations to chest compressions and outcomes. The objective of this study was to characterize the differences in chest compressions between conventional and TEE-CPR.

## Materials and methods

### Ethical approval

This Canadian multi-site study was reviewed and approved by the Research Ethics Board at the University of Saskatchewan (Beh 4008). Survey participants provided informed consent. Upon accessing the online survey, information about the study was provided and they were then given the option to participate by proceeding with the survey.

### Eligibility and recruitment

An anonymous online survey (Survey Monkey, San Francisco, USA) of healthcare providers practicing in Canada was developed to address the objectives of this study. The inclusion criteria were respondents had to be healthcare providers (nurses, physicians, residents, technicians) who had actively participated in at least one TEE-CPR event while practicing in Canada. The exclusion criterion was that respondents could not be study team members. The consent process and self-reported demographic questions were used to evaluate against the inclusion and exclusion criteria and accurately describe the respondents.

The number of providers who have participated in TEE-CPR in Canada is unknown. The team approached centres with active TEE-CPR programs that had been operating for at least a year. Of six institutions, five agreed to deploy the survey to their respective healthcare team members. The total number of Canadian healthcare providers who have participated in TEE-CPR is unknown. As such, for the sample mean to approximate a normal distribution (allowing for reliable statistical analysis), a survey sample of 30 responses was determined to be required [31]. As this was a descriptive cohort study without a comparison group, there was no effect size available for sample size calculations.

Virtual (e.g., online posters, QR codes, emails, etc.) and physical promotions (e.g., posters, flyers, etc.) were displayed to potential participants in high traffic areas at Canadian sites with established transesophageal echocardiography-guided CPR programs. Reminders to complete the survey were also sent out after CPR events at each site.

### Survey development

The survey was constructed in the context of the existing literature using quantitative and qualitative items to meet the multifaceted objectives of this research. The survey was developed by the authors in consultation with field experts. The survey was drafted, piloted, and refined into a final 34-item version (S1 File).

**Fig 1:**
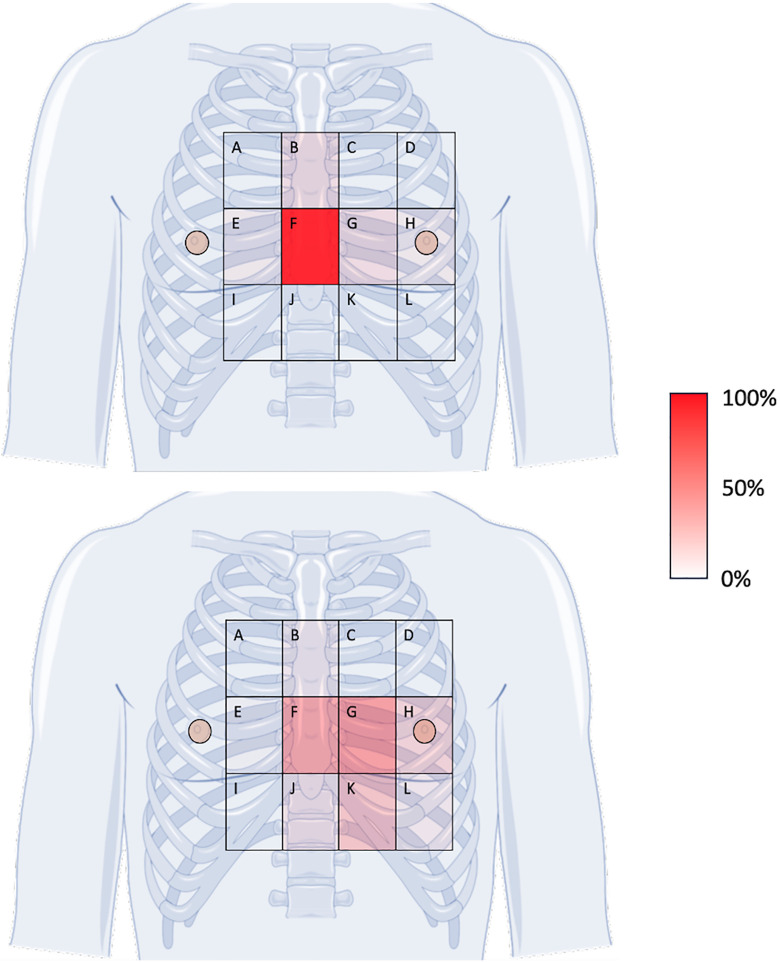
Distribution heat map of areas of compression before (top) and after (bottom) TEE-CPR was initiated. Distribution heat map (sliding scale, 100% transparency [lowest frequency] to 0% transparency [highest frequency] red shading) representing weighted mean (1^st^ choice = 1 point, 2^nd^ choice = 0.75 points, 3^rd^ choice = 0.5 points], 4^th^ choice = 0.25 points; section/total points) TEE-CPR area of compression adjustments (most common to least) as recalled by healthcare professionals.

The survey was available in English and French. The survey remained open until the *a priori* sample size of N ≥ 30 was reached. Data collection occurred from May 1^st^, 2023, through January 30, 2024. All reasonable recruitment efforts had been pursued at the conclusion of this period. Respondents were required to answer questions 1–14 in order for the survey to be considered complete, with demographic questions remaining optional. To characterize the differences in chest compressions between conventional and UG-CPR, a figure of a chest overlaid with a four by three grid was displayed (Fig 1). Using the figure, respondents were asked to first identify the conventional AOC. Then, respondents were then asked to identify, ranking from most frequent to least frequent, their recollection of the most common TEE-CPR areas of compressions for up to four areas. To cross reference findings from the visual items, respondents were also asked to describe (without a figure) in which direction TEE-CPR adjustments were made most frequently when they occurred. Providers were also asked about potential changes to chest compression dynamics including thoracic cage flexion and recoil; perceived effort, ability to remain over an alternate AOC when indicated/instructed, and the ability to return to alternative areas of compression following pulse checks.

### Quantitative analysis

A Chi-Square test was selected to examine the relationship between the pre-UG-CPR AOC (i.e., conventional CPR) and during UG-CPR. A distribution heatmap (points per space/over points across up to four choices) was generated for changes to the AOC as ranked by respondents from most common to least. For the four UG-CPR AOC choices, each selection was assigned a weighted mean (31) (most common = 1.00, 2^nd^ most common = 0.75, 3^rd^ most common = 0.50, and 4^th^ most common = 0.25). Total scores were calculated with the highest score representing the most common UG-CPR AOC. A distribution heatmap (weighted points/total points for all selections = % gradient) was generated for TEE-CPR compression AOCs [32]. For all other quantitative items, additional descriptive statistics were calculated. Means are presented as mean standard deviation. Frequencies are presented %, all percentages are calculated out of 30 unless otherwise stated.

### Qualitative analysis

A member of the research team trained in and experienced with qualitative analysis (RAM) used a pragmatic approach to inductively analyze written free text responses using standard thematic analysis methods in NVivo 14.0 (Lumivero) [33]. Codes were then categorized and grouped into themes [34].

## Results

### Demographics

N = 30 respondents from five Canadian sites described participating in UG-CPR resuscitations, 29 of whom had performed chest compressions during UG-CPR. Eighteen of the respondents described being involved in more than one TEE-CPR event, ranging from 2 to as many as 25 distinct TEE-CPR events. A description of sites, and personal and professional demographics of participants can be found in Table 1.

**Table 1:** Participant demographics.

Gender	Woman	10 (33.3%)
	Man	12 (40%)
	Prefer Not to Answer	1 (3.3%)
	No Response	7 (23.3%)
Age	Years (±SD)	37 ± 7
City	London, ON	12 (40%)
	Montreal, QB	3 (10%)
	Ottawa, ON	6 (20%)
	St.-Jean-sur-Richelieu, QB	3 (10%)
	Winnipeg, MB	3 (10%)
	No Response	3 (10%)
Roles	Physician (MD)	11 (36.7%)
	Nurse	4 (13.3%)
	Technician	4 (13.3%)
	Resident (MD)	3 (10%)
	Not specified	8 (26.7%)

Respondent (N = 30) characteristics among healthcare providers. Presented as n (%) or mean ± SD where appropriate. Percentages are rounded and may not equal 100% owing to sample size.

### Changes to chest compressions

Among healthcare providers, 96.7% respondents reported adjusting the AOC to varying extents during UG-CPR. The frequency of these UG-CPR AOC adjustments varied substantially. The majority (73.3%) indicated altering the AOC during at least half of UG-CPR events.

While 80.0% of respondents identified the lower half of the sternum (conventional landmark, item F on Fig 1) as the AOC before UG-CPR was initiated, alternate areas of compression were more common than the conventional area after initiation of UG-CPR (Pre 25;5, Post 13;17; X^2^ [1, N = 30]=10.3, *p* = 0.0013). The most common AOC after UG-CPR was initiated was to the left of the lower half of the sternum (29.7%, weighted mean). This was followed by the lower half of the sternum (27.5%), and leftward caudally (16.5%), identified as areas G, F, and K respectively on Fig 1. Combining all ranked areas, alternate areas of compression were identified as more common than the conventional area (72.5%, weighted mean, see Table 2).

**Table 2:** Areas of compression before TEE, and areas of compression in order from most common to least (up to 4 selections) including weighted mean after initiation of TEE-CPR.

AOC selected by Participants	AOC before TEE	AOC after TEE					
		Most common	2nd most common	3rd most common	Least common	Weighted Points	Weighted Mean (%)
A	0	0	0	0	0	0	0
B	2	1	0	3	0	2.5	4.2%
C	0	0	0	0	1	0.25	0.4%
D	0	0	0	0	0	0	0
E	1	0	1	0	0	0.75	1.3%
F	24	13	3	1	2	16.25	27.5%
G	2	7	11	4	1	17.5	29.7%
H	1	3	1	5	0	6.25	10.6%
I	0	0	0	0	0	0	0
J	0	2	1	2	0	3.25	5.5%
K	0	3	7	1	4	9.75	16.5%
L	0	1	1	1	1	2.5	4.2%
No selection	0	0	5	13	21	N/A	N/A
Total Participants	30	30	30	30	30	59	99.9%

AOC: Area of Compression, TEE: Transesophageal Echocardiography.

The overall reported UG-CPR AOC shift observed when using the figure of the chest was predominantly leftward (61.0%, 36/59 [weighted ranking]) and inferior (26.2% 15.5/59 [weighted ranking]). This displacement of the AOC leftward and inferiorly was similarly observed in text-based responses within the survey, with 90.0% (27/30) of respondents indicating that when moving the AOC, they moved leftward and/or inferiorly.

Differences in chest compliance (property of undergoing elastic deformation) were reported by 78.6% (22/28) of respondents, though these were described as rare by 35.7% (10/28) and as occurring some of the time by 25.0% (7/28). Despite this perceived difference in chest wall compliance, the perceived effort during TEE-CPR versus conventional compressions was reported as the same in 85.7% (24/28) of responses. There was no reported difference in chest flexion (action of bending) during TEE-CPR versus conventional CPR in 71.4% (20/28) of responses and no reported difference in chest recoil during TEE-CPR versus conventional CPR in 92.9% (26/28) of responses.

### Qualitative responses

The following themes emerged from the qualitative analysis; use of TEE for non-AOC related purposes, nuances in TEE-CPR adjustments, and compressor variation.

Beyond adjusting the AOC, respondents reported that TEE was used to monitor overall CPR efficacy (e.g., depth, rate); evaluate the etiology of the cardiac arrest; and observe for return of spontaneous circulation during resuscitation. Respondents highlighted that although TEE-CPR can be used to adjust the AOC, the other benefits of the modality should not be neglected.

Survey responses indicated that nuances during TEE-CPR in relation to the AOC can be challenging to encapsulate in a predominantly multiple-choice survey. The subtle adjustments to compressions during TEE-CPR leading to visualized improvements in hemodynamics may be challenging to illustrate in a retrospective survey.

When multiple compressors are rotating through during TEE-CPR, TEE can aid in standardizing high quality, patient-specific compressions. The use of TEE-CPR and associated real-time visual feedback guides the delivery of effective compressions.

## Discussion

This survey of healthcare providers who have participated in TEE-CPR resuscitations revealed novel information on the differences in performing chest compressions during conventional and TEE-CPR. Nearly all providers reported witnessing TEE-CPR being used to alter the AOC to areas other than the conventional landmark (the lower half of the sternum). There was a significant relationship between the use of TEE-CPR and selecting an alternate AOC, as guided by TEE visualization of compression efficacy. When indicated by TEE, leftward and inferior movements were the most common directional changes. While the widespread use of TEE-CPR is not available for the overwhelming majority of cardiac arrests in Canada, the prospective investigation and potential applications of concepts learned from TEE-CPR could improve refractory cases in the out-of-hospital and non-TEE equipped in-hospital settings.

The use of a weighted arithmetic mean allowed for inclusion of various areas of compression according to ranked frequency, though weighting was assigned without empiric data. The simultaneous consideration in the context of the qualitative responses bolsters the validity of these findings. Consistent with reports where leftward displacement of the AOC was associated with increased mean arterial pressure [35], a move leftward seemed to be the most common alternate AOC after initiation of TEE during resuscitation. This is consistent with imaging studies demonstrating the leftward and caudal (albeit heterogenous) location of the mid left ventricle [9–18].

Almost 75% of respondents reported adjusting where they performed compressions in ≥50% of cases. This finding is also consistent with recent case series in which the use of TEE-CPR in the emergency department resulted in a change from the conventional-CPR AOC [28,29] as often as ~50% of the time [28]. Similarly, a case series demonstrated consistent hemodynamic improvement across five patients when using transthoracic ultrasound to guide chest compression location to improve ventricular compression during CPR [36]. However, these studies do not provide data regarding the exact location of the novel AOCs [28,29,37].

The interpretation of TEE images by those performing compressions, with subsequent autonomous modification of compressions, heralds a novel form of chest compression monitoring and feedback. If health care providers with minimal training in TEE imaging are able to adjust their compressions using these images, it is possible that such interpretations and adjustments could be achieved through automated ultrasound in the prehospital setting as well [38].

The effects on thoracoabdominal injury when altering the AOC away from the conventional CPR location remain unclear [24–29]. Thoracoabdominal injuries are common and well-documented to occur from conventional CPR [39–43]. How injury patterns may differ when the AOC is altered during TEE-CPR remains unclear as current studies focus on TEE-transducer-related injury and not injuries associated with alternate AOCs [44]. Some experimental reports suggest there may be an increased risk of flail chest segments and associated injuries [45,46]. Prospective mapping of areas of compression during TEE-CPR events should also include attention to potential changes in injury patterns as identified during subsequent advanced imaging [35]. Such research would help inform whether a novel AOC landmark should be entertained, either as an alternate in certain patient groups or as a next best AOC when conventional CPR efforts fail to return spontaneous circulation in OHCA.

### Limitations

As Canada has relatively few UG-CPR enabled sites, and response rates to online surveys tend to be low, the sample size was small. To reach 30 responses, the survey remained open beyond the original study period of three months to a total of eight months. Furthermore, as this was a survey asking participants to recall past events, recall bias was an inherent limitation. However, there is evidence that healthcare providers do have reasonably high recall accuracy following resuscitation events [47]. While it is possible that participants from each site were reflecting on some of the same events, it appears that most (18/30 respondents) had attended a range of 2–25 distinct TEE-CPR events, thus increasing the total number of TEE-CPR events captured by this survey beyond 50. Though adjustments to the AOC appear to be common, the figures herein may not have been adequately sensitive to capture the nuances of finer UG-CPR compression adjustments (changes to not only the AOC but also direction of downward thrust leftward/rightward or cranial/caudal, as well as changes from one compression cycle to another). Future research should aim to validate these findings prospectively to determine if the changes to AOC described here are accurate. Finally, as a retrospective survey, this study design does not inform when changes to the AOC were associated with changes in patient outcomes. Future prospective studies should match changes to the AOC to clinical outcomes. If certain changes are associated with favorable outcomes, such changes to compressions might be generalizable to OHCA patients attended to by first responders prior to any potential deployment of UG-CPR.

## Conclusion

Healthcare providers, with guidance from TEE, are performing chest compressions at alternative areas to the conventional AOC. The general nature of those adjustments is reported to be leftward and caudal, aligning with recent research that indicates chest compressions over the left ventricle increase CPR efficacy. Although the exact nature of UG-CPR chest compression adjustments cannot be fully elucidated through the methods employed herein, evidence that alternative areas of compression are already being used enables more robust investigations.

## Supporting information

S1 File Survey (English). (PDF)

S2 File Survey Responses. English and French.
(PDF)
