# Supplementary material for: Transesophageal echocardiography during cardiopulmonary resuscitation is associated with alternate areas of compression: Analysis of healthcare provider experiences with potential implications for conventional compressions
Source: PLoS One. 2026 Jan 20;21(1):e0339974. doi: 10.1371/journal.pone.0339974 (PMC12818596; doi:10.1371/journal.pone.0339974)
Supplement: S1 File — (PDF) [file pone.0339974.s001.pdf]

Participant Consent Form

You are invited to participate in a research study entitled: Performing Chest Compressions under Transesophageal Echocardiographic (TEE) Guidance: Analysis of Healthcare Provider Experiences.

[redacted]

As you are a healthcare provider working at a site with TEE-guide cardiac arrest care, we are inviting you to answer this brief anonymous questionnaire. We are interested in understanding how the use of TEE during CPR impacts the resuscitation experience. The research team is particularly interested in the impacts of TEE-guidance on those performing chest compressions as they are now moving the area of compression off or away from the established anatomic-landmark (mid chest, lower half of the sternum). This is a research study where your participation may lead to our research team identifying a novel landmark for chest compressions during cardiopulmonary resuscitation (CPR).

Procedures:

We have developed an anonymous online survey for healthcare providers who have participated in TEE-guided CPR. We are asking providers to describe their experiences performing chest compressions including initial chest compression location, optimal TEE-guided chest compression location, differences in chest wall compliance, and if it was difficult to remain/return over the area after pulse/rhythm checks. The Survey will take 5-10 minutes.

Funded by:

[redacted]

Potential Risks:

Participant information and data will be kept anonymous. Participation will require the providers to reflect on previous experiences performing chest compressions during cardiac arrest. We acknowledge the potential stress that reflection on chest compressions can bring and in instances of strong emotional reactions, please reach out to your local healthcare provider wellness programs such as the Centre for Addiction and Mental Health crisis resource page (https://www.camh.ca/en/health-info/crisis-resources).

City/Province Specific Information

Manitoba
Please vist https://www.gov.mb.ca/mh/mh/crisis.html.

Ontario
Please reach out to the employee assistance program, your professional organization support program, or ConnexOntario.

London, Ontario
Staff Support is a branch of Where Wellness Works committed to supporting the emotional wellbeing and professional fulfillment of staff, leaders and physicians at LHSC.
Staff Support is available 24 hours, 7 days a week
Dial extension 18182 to page us.
Or call 519.685.8500 extension 18182.

Potential Benefits:

The findings of this survey will help inform future research exploring an alternative/improved area of compression landmark for both in-hospital- and out-of-cardiac arrest. Data and synthesis will contribute to this overall effort which is aiming toward a randomized control trial of standard vs left-sided chest compressions in cardiac arrest.

Confidentiality:

Although the data from this research project will be published and presented at conferences, the survey data will be kept anonymous with findings reported in aggregate form so that it will not be possible to identify individuals. Direct quotations will not be used, instead we will use paraphrasing to convey key messages and findings. No details related to institutional affiliation of any respondent will be included. This survey is hosted by Survey Monkey. Your data will be stored in facilities hosted in Canada. Please see the following for more information on the Survey Monkey Privacy Policy”.

__Storage of Data__:

The Principal Investigator will be responsible for the security and storage of the data. Electronic data will be stored on the Principal Investigator’s password-protected computer during analyses but moved to a USask system for long-term storage (OneDrive). Data will be stored for the minimum required storage period of five years post-publication. Once the data is no longer required and following the required storage period, the data will be destroyed beyond recovery. These too will be destroyed once no longer required after the storage period. The Principal Investigator will be responsible for the security and storage of the data.

__Right to Withdraw__:

Participation in this survey is voluntary.
You can decide not to participate at any time by closing your browser, or choose not to answer any questions you do not feel comfortable with. Survey responses will remain anonymous. Since the survey is anonymous, once it is submitted it cannot be removed.
Whether you choose to participate or not will have no effect on your position (e.g., employment, academic status, access to services) or how you will be treated.

__Follow up__:

To obtain results from the study (estimated to be available fall of 2023) please email the Principal Investigator p.olszynski@usask.ca. We also intend to publish our findings and will make an abstract available at www.sasksonic.com
We advise you download a copy of this consent form for your records.

__Questions or Concerns__:

Contact the researcher(s) using the information at the top of page 1.

By completing and submitting this questionnaire, **your free and informed consent is implied** and indicates that you understand the above conditions of participation in this study.

Introduction

1. Please indicate which province or territory you reside in

- Alberta
- British Columbia
- Manitoba
- New Brunswick
- Newfoundland and Labrador
- Nova Scotia
- Ontario
- Prince Edward Island
- Quebec
- Saskatachewan
- Northwest Territories
- Nunavut
- Yukon
- Prefer not to anwser

2. Optional : What city do you work in?

* 3. Have you attended a code blue (cardiac arrest resuscitation) where a practitioner used trasesophageal echocardiography (TEE)? If not, we ask that you discontinue the survey at this time and thank you for your consideration.

- Yes
- No

* 4. Have you performed chest compressions under guidance from TEE?

- Yes
- No

* 5. Before TEE is being used to guide compression, were chest compressions performed on the lower half of the sternum/centre of the chest as shown by the image below (box F)

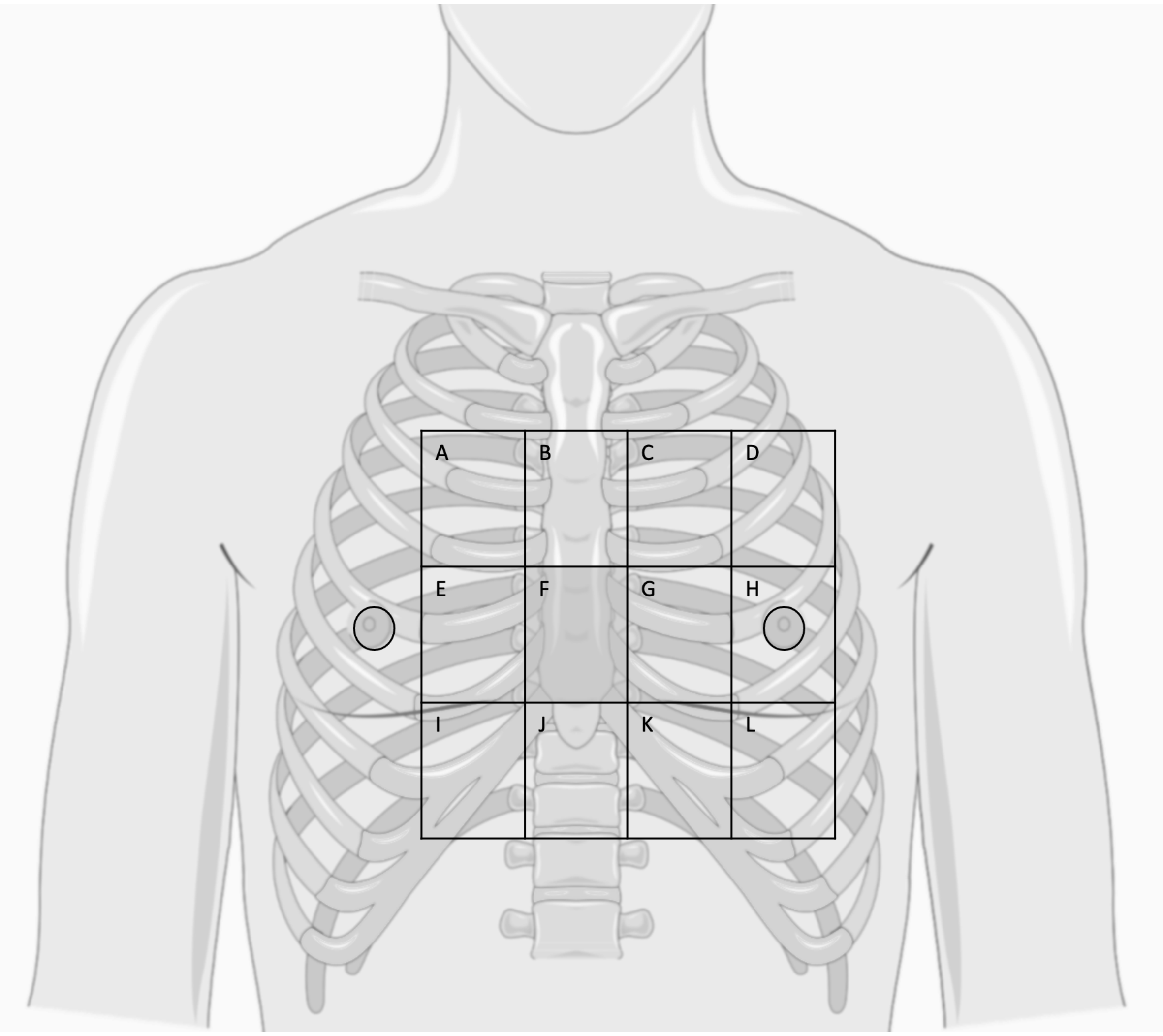

- ◯ Yes
- ◯ No

* 6. How many TEE-guided codes have you attended?

* 7. How often does the use of TEE during cardiac arrest result in compressions being performed on a different part of the chest?

- ◯ Never (0%)
- ◯ Rarely
- ◯ Sometimes (~50%)
- ◯ Often
- ◯ Always (100%)

The next series of questions ask specifically when and how you were told to adjust chest compressions.

* 8. When TEE is in use, are you typically instructed by a member of the team to move where you are doing chest compressions?

- ◯ Yes
- ◯ No

* 9. If you typically moved away from the centre of the chest, where did you move to (check all that apply)?

- ☐ A) Patient’s left chest (towards left nipple)
- ☐ B) Inferiorly (towards legs)
- ☐ C) Patient’s right chest (towards right nipple)
- ☐ D) Upwards (towards head)

* 10. Typically, were you given verbal instructions to move to a new area of compression?

- ◯ Yes
- ◯ No

* 11. Typically, were you provided verbal feedback/instructions from the team to guide you when you were at the correct area of compression?

- ◯ Yes
- ◯ No

* 12. Typically, were you provided visual feedback from the echocardiography screen to guide your compressions?

- ◯ Yes
- ◯ No

* 13. Using the corresponding zone letter, what area do you typically start CPR in before TEE?

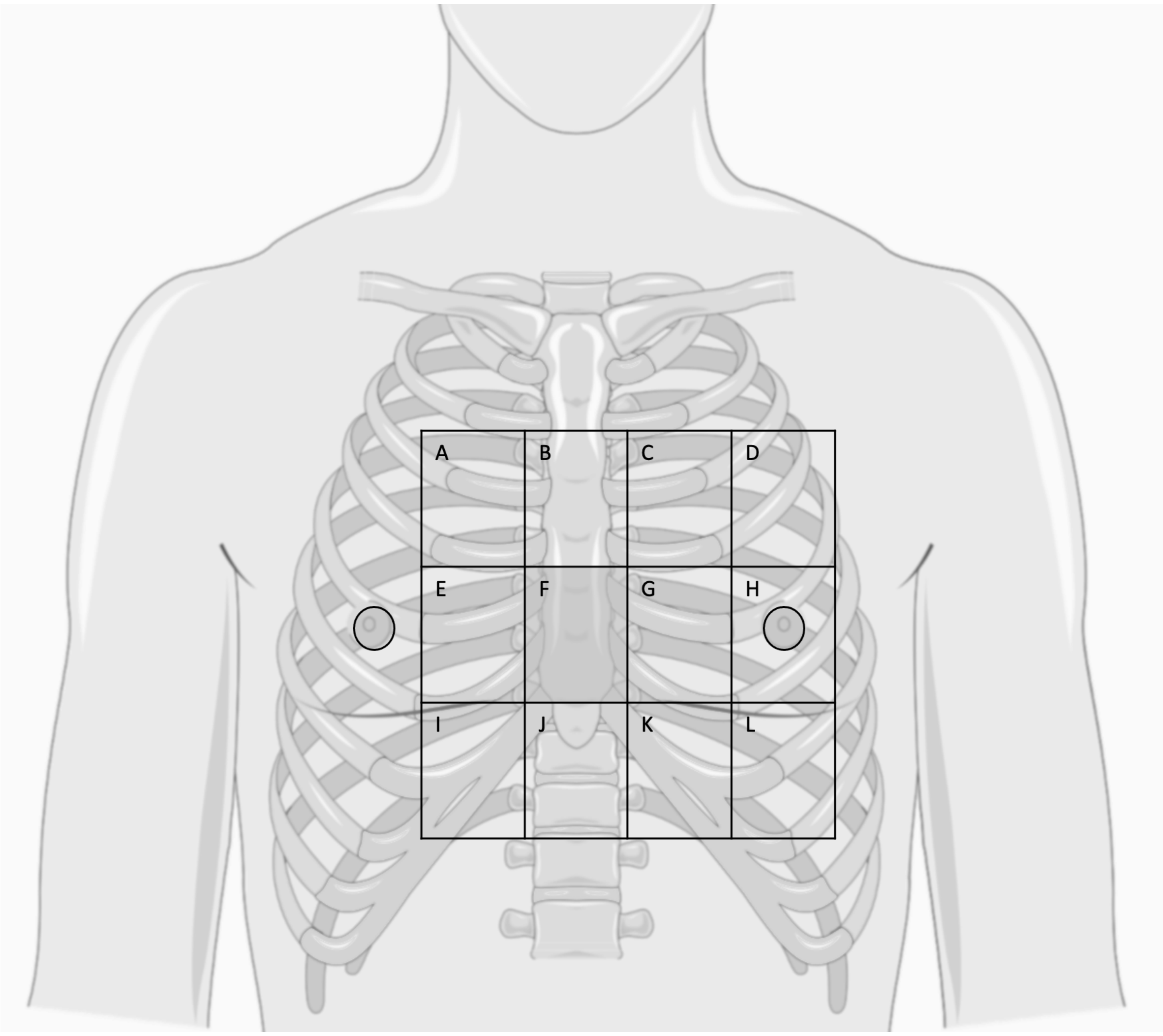

* 14. Using the corresponding zone letters, please list in order of frequency (1 being most common) the top 4 locations you typically perform compressions after TEE is initiated. If you use less than 4 locations, leave the empty boxes blank.

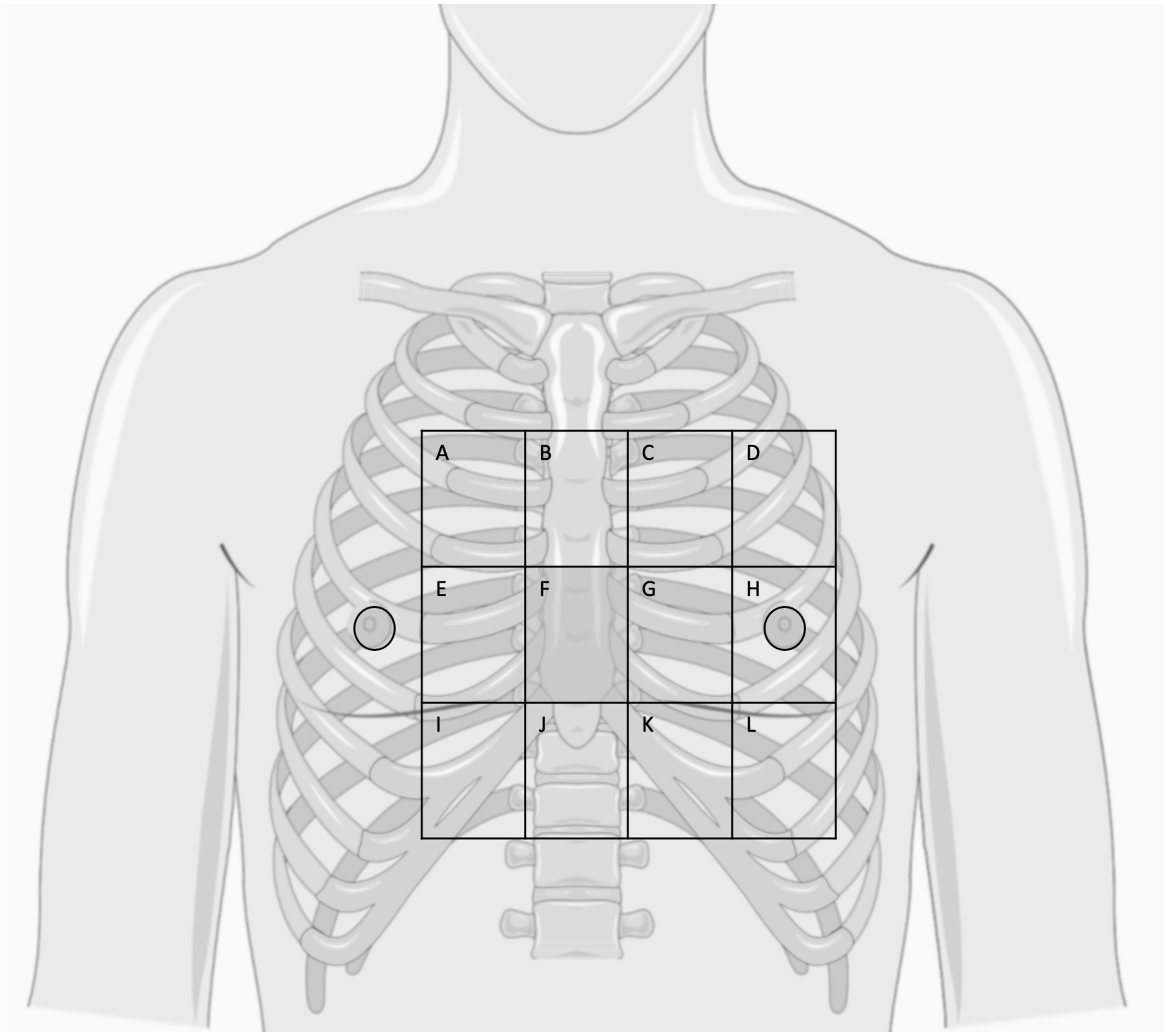

1. (most common) [ ]

2. [ ]

3. [ ]

4. [ ]

The next series of questions will ask what it feels like to perform compressions off the centre of chest/lower half of sternum.

* 15. Did you perceive a difference in chest compliance (examples include flex or recoil of the chest wall)?

- ◯ Never
- ◯ Rarely
- ◯ Sometimes
- ◯ Usually
- ◯ Always

* 16. Was there a difference in chest flexion (resistance to the chest being compressed during compressions)?

- ◯ Downward chest compression was more difficult
- ◯ Downward chest compression was easier
- ◯ Downward chest compression was the same
- ◯ Not Listed (please specify)
- ◯ No difference noticed

* 17. Was there a difference in chest recoil (degree to which the chest returns to non-compressed/anatomic shape)?

- ☐ Less recoil
- ☐ About the same recoil
- ☐ More recoil
- ☐ Not Listed (please specify)
- ☐ No difference noticed

* 18. Once identified, is the TEE-guided location of compression typically challenging to maintain between pulse/rhythm checks?

- ◯ Never
- ◯ Rarely
- ◯ Sometimes
- ◯ Often
- ◯ Always

* 19. During chest compressions, are you typically able to maintain compressions over the TEE-guided location?

- Never
- Rarely
- Sometimes
- Often
- Always

20. What typically were the challenges that lead to not being able to perform compressions in the adjusted area of the chest?
For example, less structure, no land mark, ergonomics, etc.

* 21. Rate your perceived effort of performing chest compressions off the lower half of sternum/centre of chest?

	Much less effort	Less effort	Same effort (neutral)	More effort	Much more effort
Response	○	○	○	○	○

22. Are you ever shown images from the TEE machine to help you guide your compression quality (rate, depth, location, etc.)?

- Never
- Rarely
- Sometimes
- Usually
- Always

* 23. What is your current job title in the context of performing resuscitation?

- Staff Physician
- Resident Physician
- Physician’s Assistant
- Registered Nurse
- Licensed Practical Nurse
- Applied Care Aid
- Ultrasound Technician
- Respiratory Therapist
- Student (please specify in text box)
- Not Listed (please specify in text box)

If you are a student or your job title is not listed, please specify below:

* 24. How many years have you been in practice (round to the nearest year)?

0 70

* 25. How many years experience do you have performing resuscitation in your current role (round to nearest year)?

0 70

* 26. In an average year, aproximately how many codes (cardiopulmonary resuscitations) do you attend?

* 27. Over the course of your entire career, aproximately how many codes (cardiopulmonary resuscitations) have you attended?

* 28. How old are you?

18 100

* 29. What gender do you identify as?

- ◯ Women
- ◯ Man
- ◯ Non-Binary/Third Gender
- ◯ Not listed (please specify)

[ ]

- ◯ Prefer not to answer

* 30. To examine the potential influence of sexual dimorphism (physiological differences in form between individuals of different sex such as differences in the average width of the palm between sexes), do you have lived experience as a trans person (meaning your gender identity does not align with your gender assigned at birth)?

- ◯ Yes
- ◯ No
- ◯ Prefer not to answer

* 31. Do you identify as a person currently living with a physical disability?

- ◯ Yes
- ◯ No
- ◯ Prefer not to anwser

* 32. What is your cultural background?
Choose all that apply.

- ☐ African
- ☐ European
- ☐ East Asian
- ☐ First Nations or Indigenous
- ☐ Hispanic or Latinx
- ☐ Middle Eastern
- ☐ South Asian
- ☐ South East Asian
- ☐ Not Listed (please specify)

[ ]

- ☐ Prefer not to anwser

33. Please specify your First Nations or Indigenous cultural background

34. Do you have any additional comments you would like to share regarding your experience performing TEE-guided chest compressions before we move to the next part of the survey?

35. If you are interested in participating in a follow-up interview, please provide you name and email address:

Name

Email Address

Thank you for completing the survey!
