# Supplementary material for: Transesophageal echocardiography during cardiopulmonary resuscitation is associated with alternate areas of compression: Analysis of healthcare provider experiences with potential implications for conventional compressions
Source: PLoS One. 2026 Jan 20;21(1):e0339974. doi: 10.1371/journal.pone.0339974 (PMC12818596; doi:10.1371/journal.pone.0339974)
Supplement: S2 File — English and French. (PDF) [file pone.0339974.s002.pdf]

Q1 Please indicate which province or territory you reside in

Answered: 37 Skipped: 1

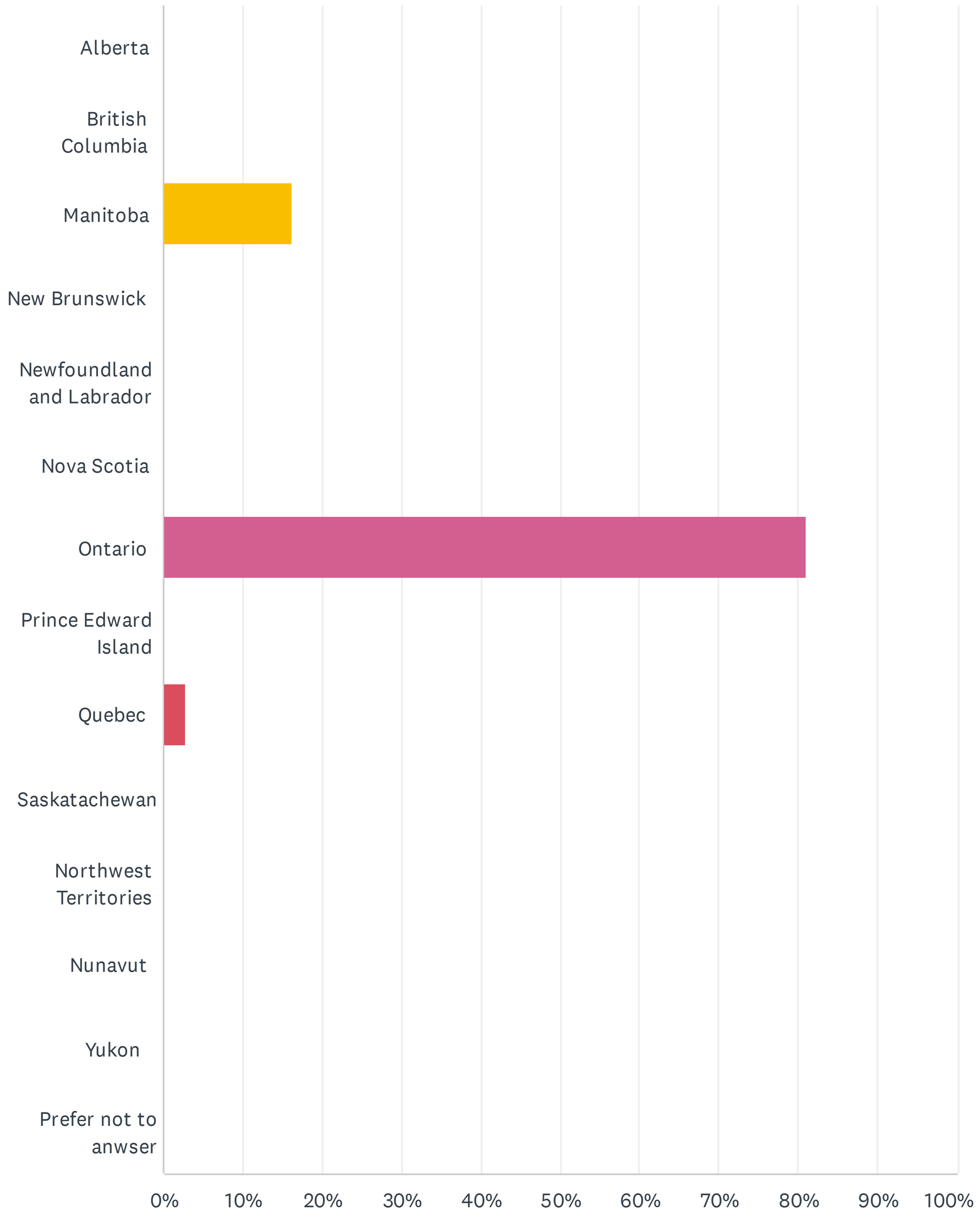

ANSWER CHOICES	RESPONSES	
Alberta	0.00%	0
British Columbia	0.00%	0
Manitoba	16.22%	6
New Brunswick	0.00%	0
Newfoundland and Labrador	0.00%	0
Nova Scotia	0.00%	0
Ontario	81.08%	30
Prince Edward Island	0.00%	0
Quebec	2.70%	1
Saskatachewan	0.00%	0
Northwest Territories	0.00%	0
Nunavut	0.00%	0
Yukon	0.00%	0
Prefer not to anwser	0.00%	0
TOTAL		37

Q2 Optional : What city do you work in?

Answered: 32 Skipped: 6

Q3 Have you attended a code blue (cardiac arrest resuscitation) where a practitioner used trasesophageal echocardiography (TEE)? If not, we ask that you discontinue the survey at this time and thank you for your consideration.

Answered: 36 Skipped: 2

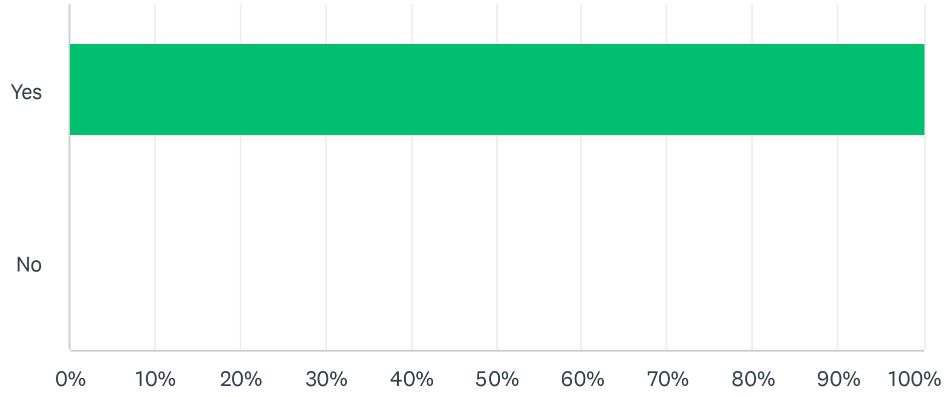

ANSWER CHOICES	RESPONSES	
Yes	100.00%	36
No	0.00%	0
TOTAL		36

Q4 Have you performed chest compressions under guidance from TEE?

Answered: 36 Skipped: 2

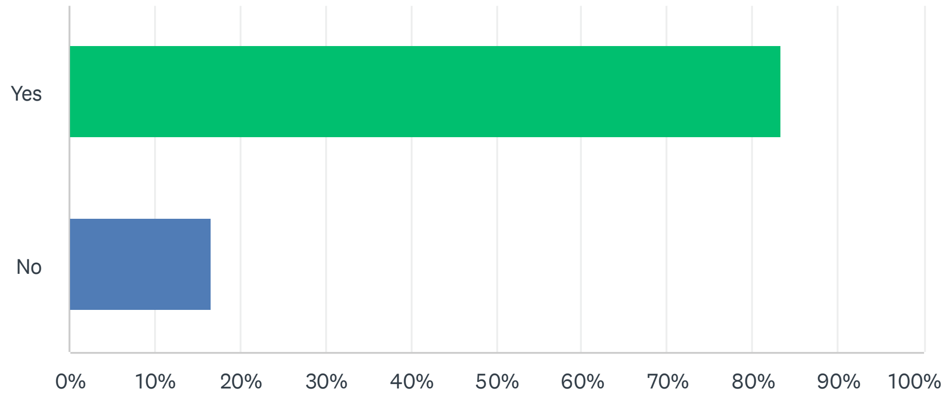

ANSWER CHOICES	RESPONSES	
Yes	83.33%	30
No	16.67%	6
TOTAL		36

Q5 Before TEE is being used to guide compression, were chest compressions performed on the lower half of the sternum/centre of the chest as shown by the image below (box F)

Answered: 31 Skipped: 7

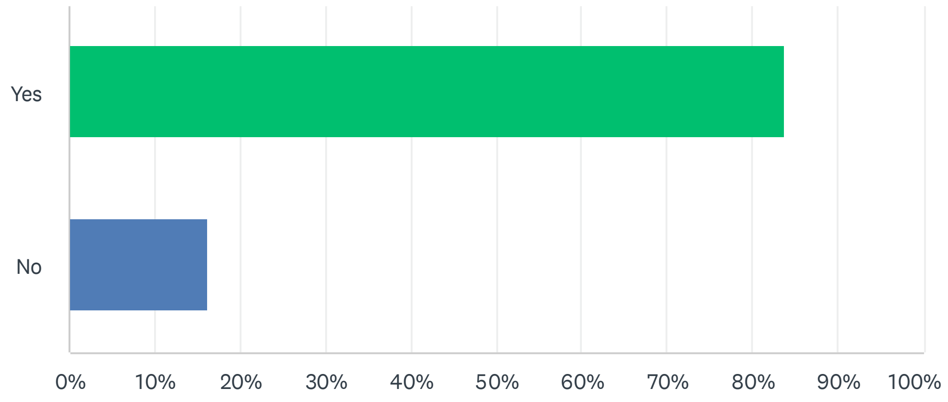

ANSWER CHOICES	RESPONSES	
Yes	83.87%	26
No	16.13%	5
TOTAL		31

Q6 How many TEE-guided codes have you attended?

Answered: 23 Skipped: 15

Q7 How often does the use of TEE during cardiac arrest result in compressions being performed on a different part of the chest?

Answered: 31 Skipped: 7

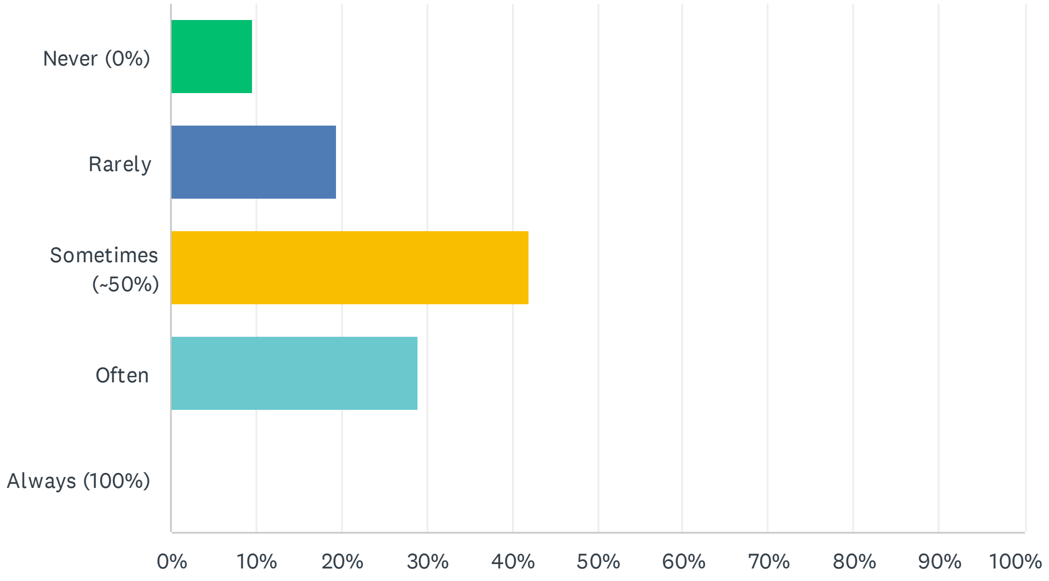

ANSWER CHOICES	RESPONSES	
Never (0%)	9.68%	3
Rarely	19.35%	6
Sometimes (~50%)	41.94%	13
Often	29.03%	9
Always (100%)	0.00%	0
TOTAL		31

Q8 When TEE is in use, are you typically instructed by a member of the team to move where you are doing chest compressions?

Answered: 24   Skipped: 14

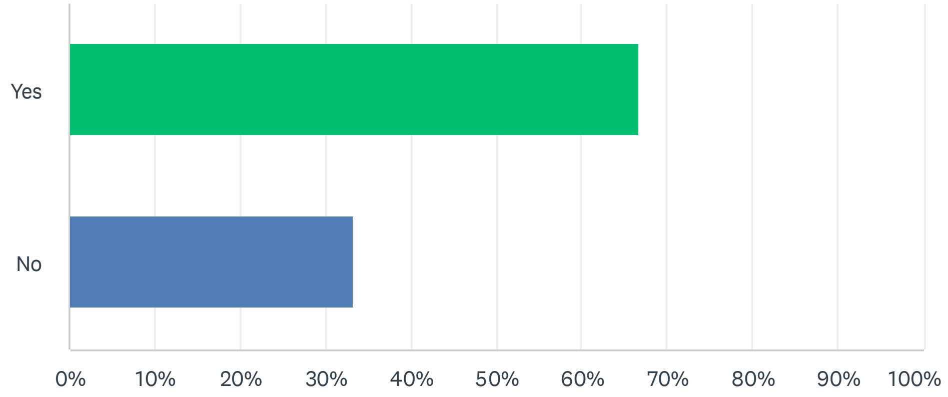

ANSWER CHOICES	RESPONSES	
Yes	66.67%	16
No	33.33%	8
TOTAL		24

Q9 If you typically moved away from the centre of the chest, where did you move to (check all that apply)?

Answered: 24 Skipped: 14

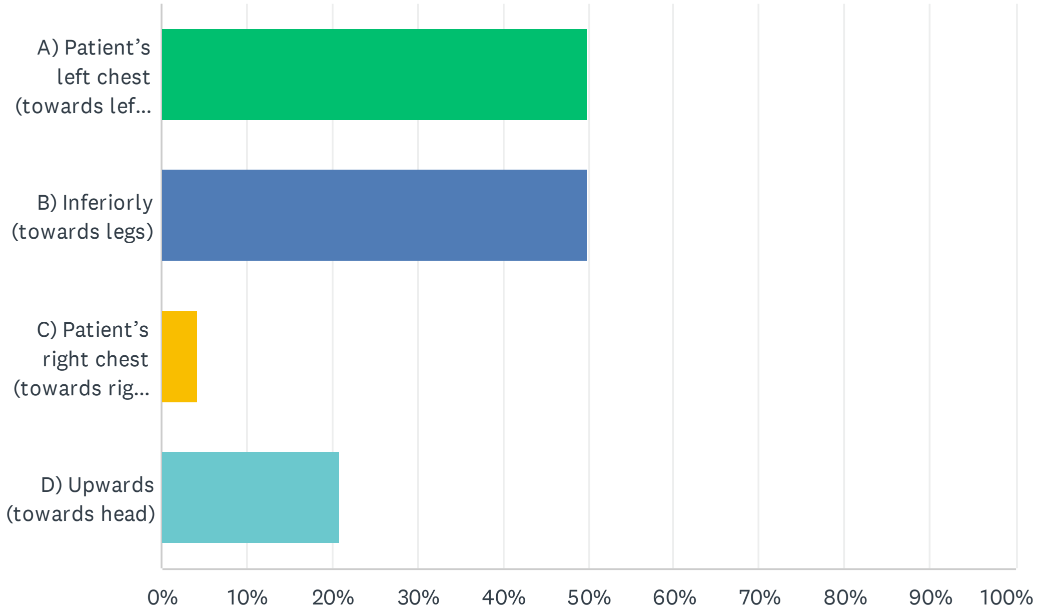

ANSWER CHOICES	RESPONSES	
A) Patient's left chest (towards left nipple)	50.00%	12
B) Inferiorly (towards legs)	50.00%	12
C) Patient's right chest (towards right nipple)	4.17%	1
D) Upwards (towards head)	20.83%	5
Total Respondents: 24		

Q10 Typically, were you given verbal instructions to move to a new area of compression?

Answered: 24 Skipped: 14

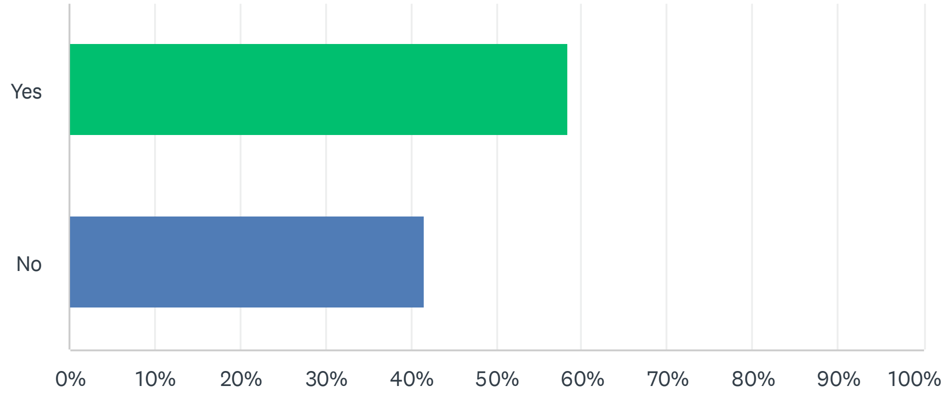

ANSWER CHOICES	RESPONSES	
Yes	58.33%	14
No	41.67%	10
TOTAL		24

Q11 Typically, were you provided verbal feedback/instructions from the team to guide you when you were at the correct area of compression?

Answered: 24 Skipped: 14

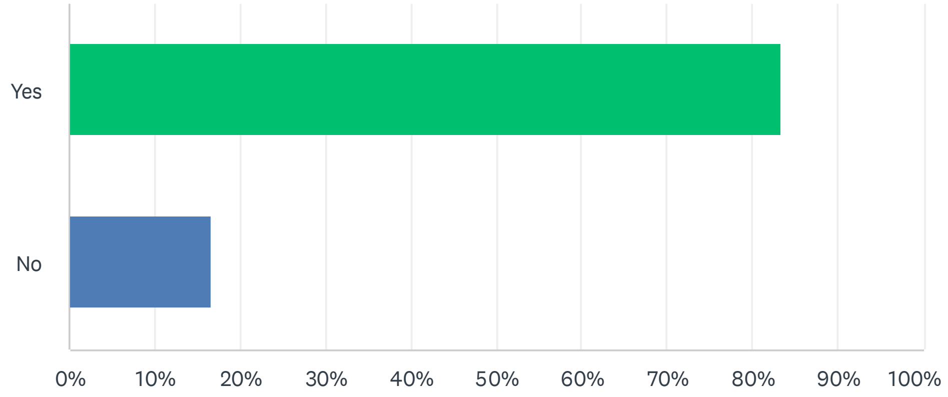

ANSWER CHOICES	RESPONSES	
Yes	83.33%	20
No	16.67%	4
TOTAL		24

Q12 Typically, were you provided visual feedback from the echocardiography screen to guide your compressions?

Answered: 24 Skipped: 14

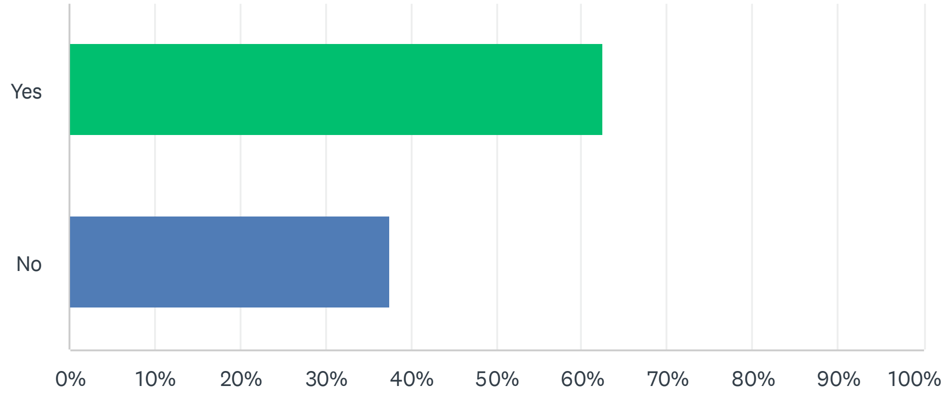

ANSWER CHOICES	RESPONSES	
Yes	62.50%	15
No	37.50%	9
TOTAL		24

Q13 Using the corresponding zone letter, what area do you typically start CPR in before TEE?

Answered: 24 Skipped: 14

Q14 Using the corresponding zone letters, please list in order of frequency (1 being most common) the top 4 locations you typically perform compressions after TEE is initiated. If you use less than 4 locations, leave the empty boxes blank.

Answered: 24 Skipped: 14

ANSWER CHOICES	RESPONSES	
1. (most common)	100.00%	24
2.	83.33%	20
3.	62.50%	15
4.	33.33%	8

Q15 Did you perceive a difference in chest compliance (examples include flex or recoil of the chest wall)?

Answered: 23 Skipped: 15

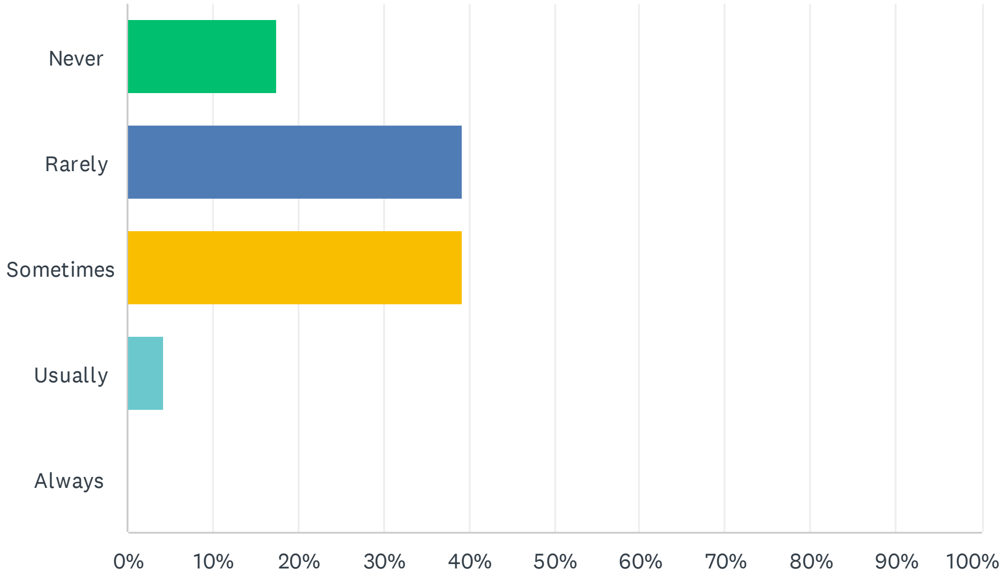

ANSWER CHOICES	RESPONSES	
Never	17.39%	4
Rarely	39.13%	9
Sometimes	39.13%	9
Usually	4.35%	1
Always	0.00%	0
TOTAL		23

Q16 Was there a difference in chest flexion (resistance to the chest being compressed during compressions)?

Answered: 23 Skipped: 15

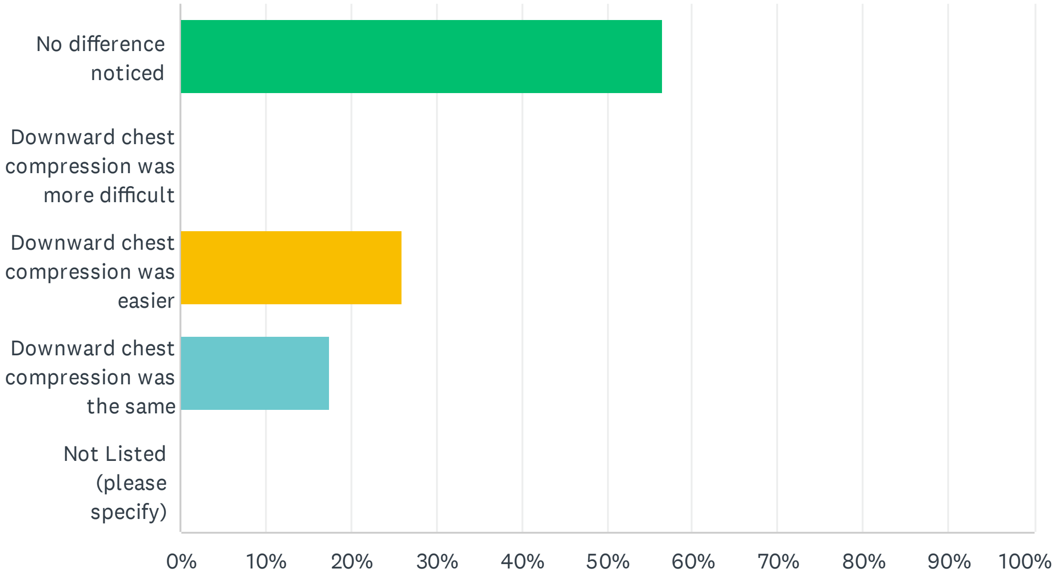

ANSWER CHOICES	RESPONSES	
No difference noticed	56.52%	13
Downward chest compression was more difficult	0.00%	0
Downward chest compression was easier	26.09%	6
Downward chest compression was the same	17.39%	4
Not Listed (please specify)	0.00%	0
TOTAL		23

Q17 Was there a difference in chest recoil (degree to which the chest returns to non-compressed/anatomic shape)?

Answered: 23 Skipped: 15

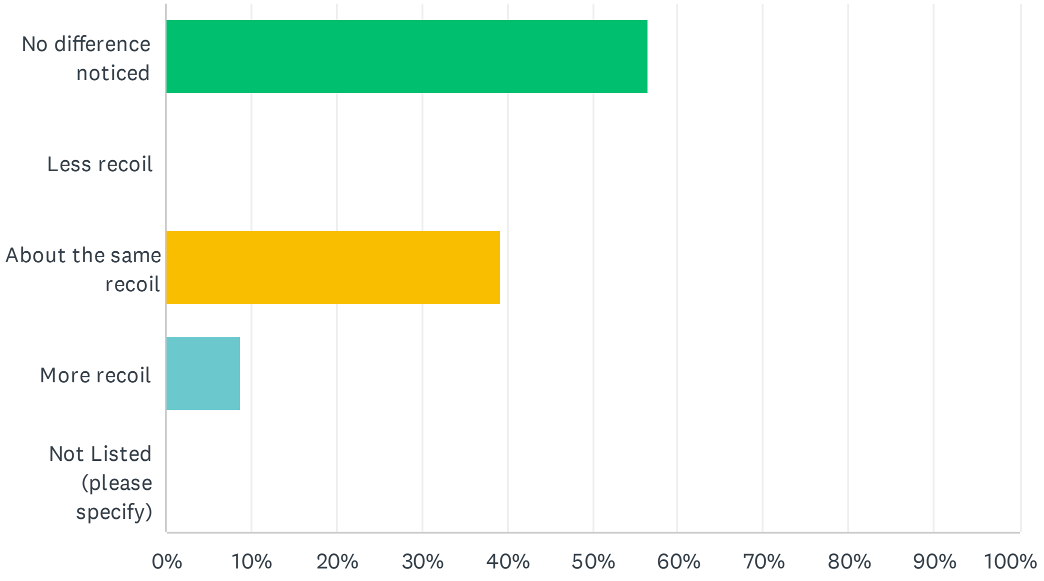

ANSWER CHOICES	RESPONSES	
No difference noticed	56.52%	13
Less recoil	0.00%	0
About the same recoil	39.13%	9
More recoil	8.70%	2
Not Listed (please specify)	0.00%	0
Total Respondents: 23		

Q18 Once identified, is the TEE-guided location of compression typically challenging to maintain between pulse/rhythm checks?

Answered: 23 Skipped: 15

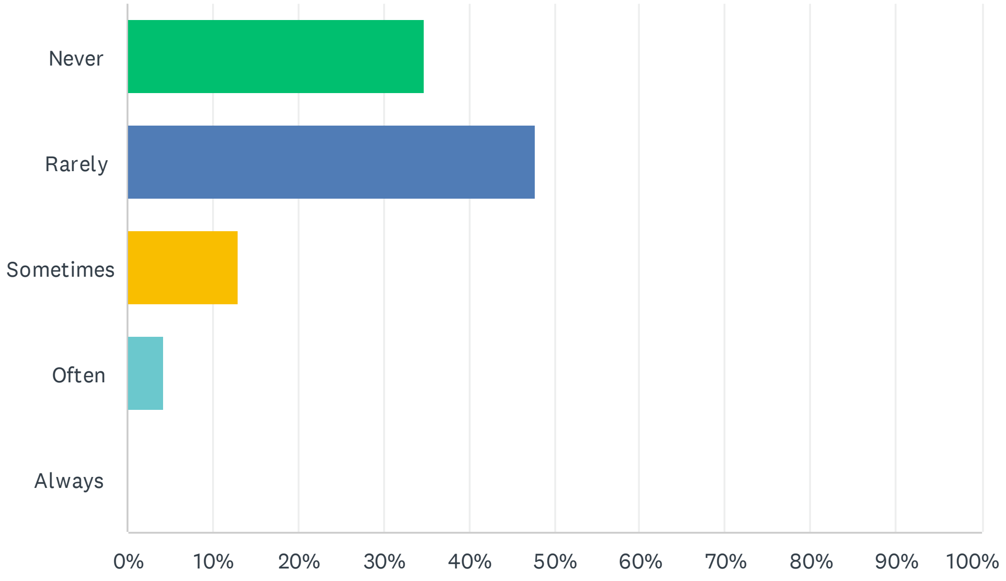

ANSWER CHOICES	RESPONSES	
Never	34.78%	8
Rarely	47.83%	11
Sometimes	13.04%	3
Often	4.35%	1
Always	0.00%	0
TOTAL		23

Q19 During chest compressions, are you typically able to maintain compressions over the TEE-guided location?

Answered: 23 Skipped: 15

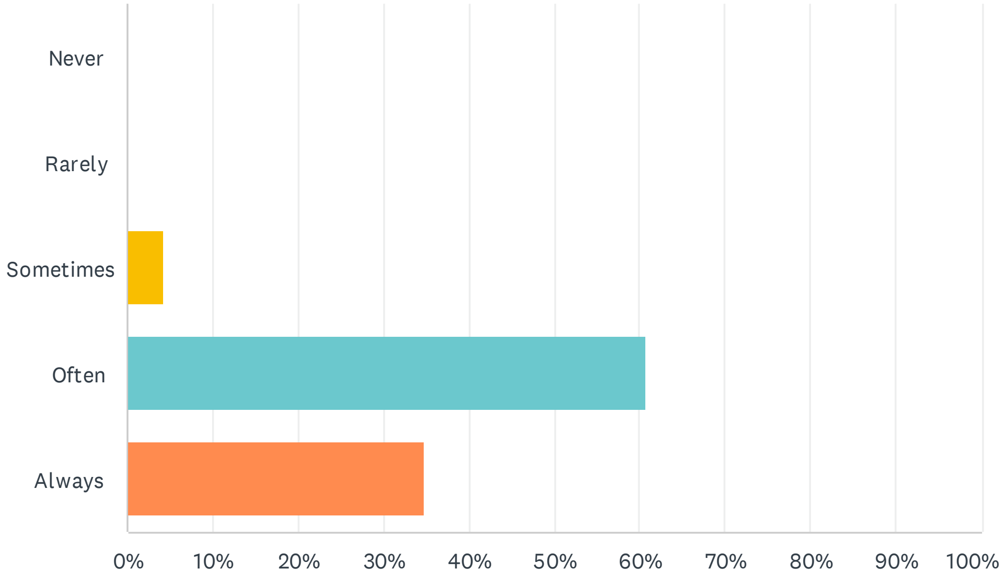

ANSWER CHOICES	RESPONSES	
Never	0.00%	0
Rarely	0.00%	0
Sometimes	4.35%	1
Often	60.87%	14
Always	34.78%	8
TOTAL		23

Q20 What typically were the challenges that lead to not being able to perform compressions in the adjusted area of the chest?For example, less structure, no land mark, ergonomics, etc.

Answered: 15 Skipped: 23

Q21 Rate your perceived effort of performing chest compressions off the lower half of sternum/centre of chest?

Answered: 21 Skipped: 17

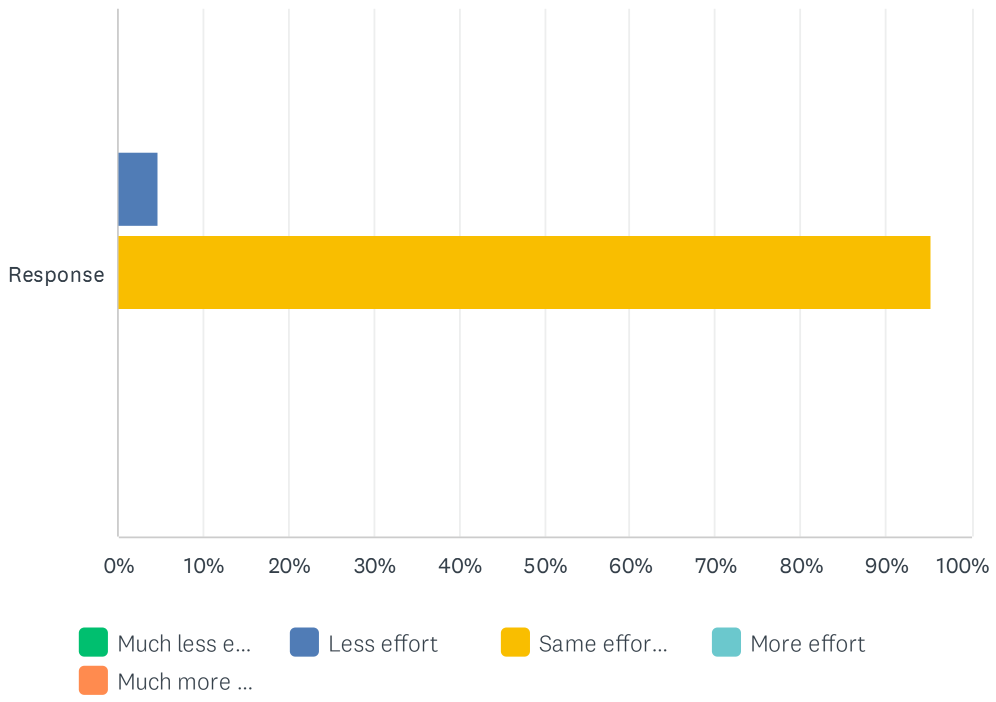

	MUCH LESS EFFORT	LESS EFFORT	SAME EFFORT (NEUTRAL)	MORE EFFORT	MUCH MORE EFFORT	TOTAL	WEIGHTED AVERAGE
Response	0.00%<br>0	4.76%<br>1	95.24%<br>20	0.00%<br>0	0.00%<br>0	21	1.00

Q22 Are you ever shown images from the TEE machine to help you guide your compression quality (rate, depth, location, etc.)?

Answered: 21 Skipped: 17

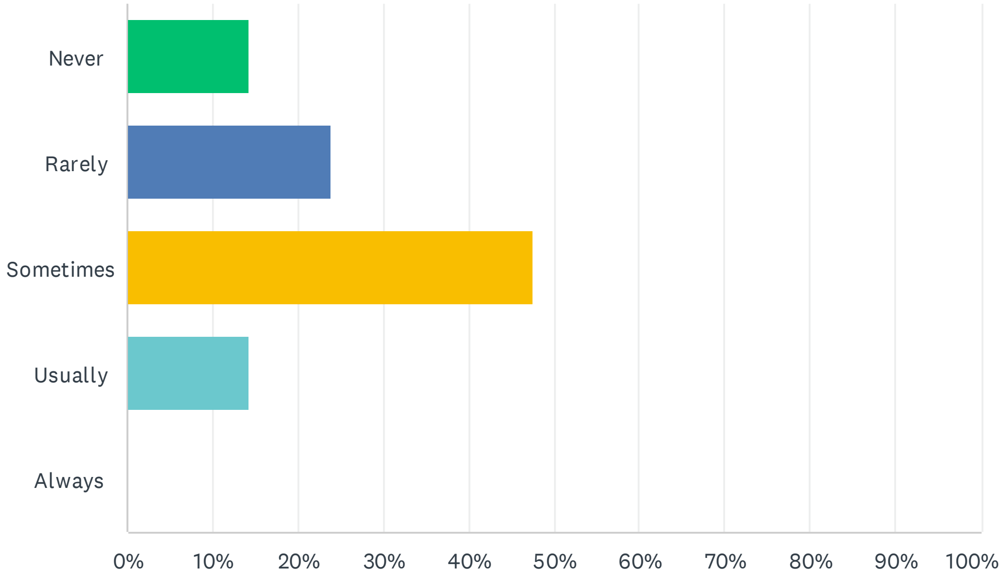

ANSWER CHOICES	RESPONSES	
Never	14.29%	3
Rarely	23.81%	5
Sometimes	47.62%	10
Usually	14.29%	3
Always	0.00%	0
TOTAL		21

Q23 What is your current job title in the context of performing resuscitation?

Answered: 20 Skipped: 18

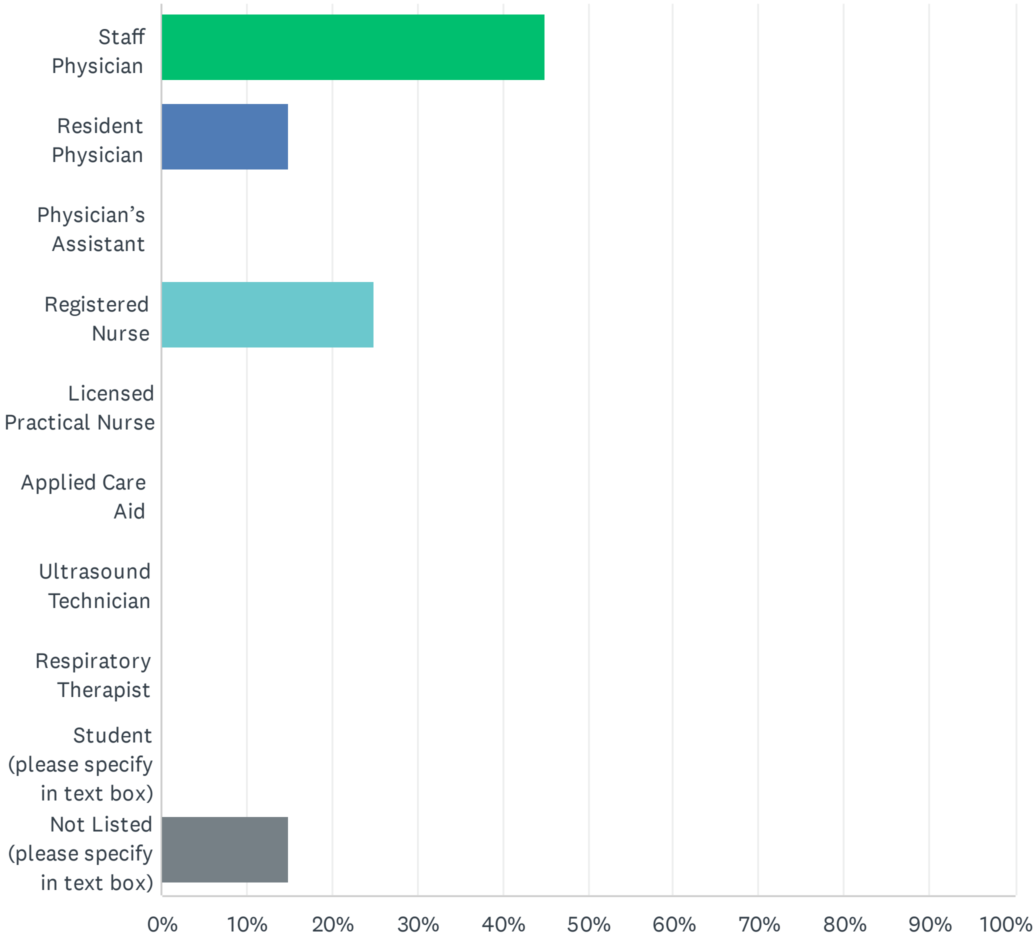

ANSWER CHOICES	RESPONSES	
Staff Physician	45.00%	9
Resident Physician	15.00%	3
Physician’s Assistant	0.00%	0
Registered Nurse	25.00%	5
Licensed Practical Nurse	0.00%	0
Applied Care Aid	0.00%	0
Ultrasound Technician	0.00%	0
Respiratory Therapist	0.00%	0
Student (please specify in text box)	0.00%	0
Not Listed (please specify in text box)	15.00%	3
TOTAL		20

Q24 How many years have you been in practice (round to the nearest year)?

Answered: 20 Skipped: 18

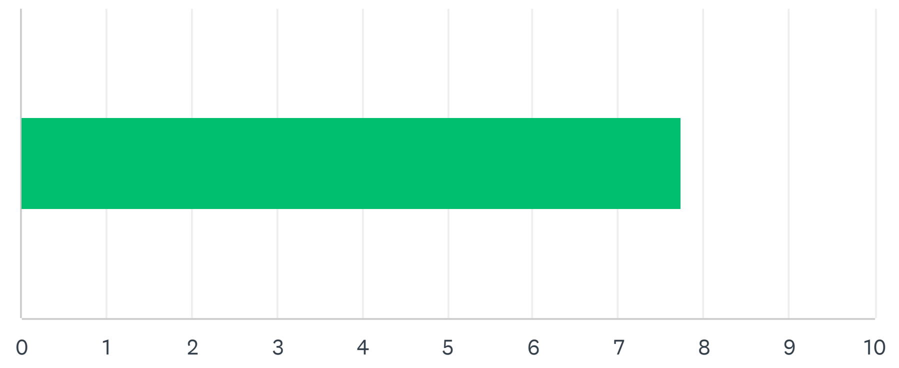

ANSWER CHOICES	AVERAGE NUMBER	TOTAL NUMBER	RESPONSES
	8	155	20
Total Respondents: 20			

Q25 How many years experience do you have performing resuscitation in your current role (round to nearest year)?

Answered: 20 Skipped: 18

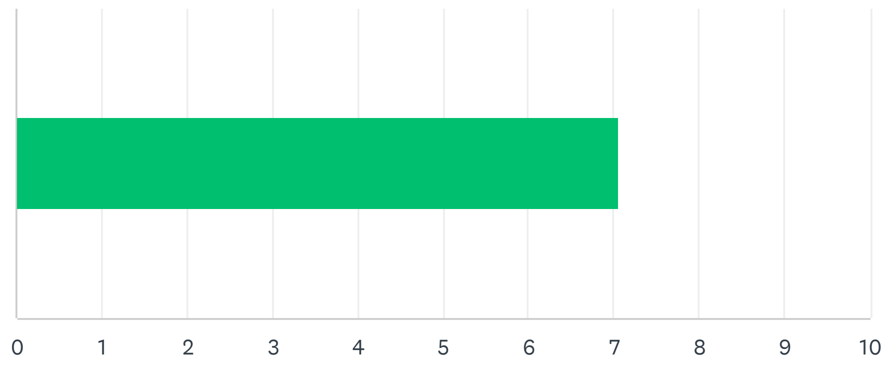

ANSWER CHOICES	AVERAGE NUMBER	TOTAL NUMBER	RESPONSES
	7	141	20
Total Respondents: 20			

Q26 In an average year, aproximately how many codes (cardiopulmonary resuscitations) do you attend?

Answered: 20 Skipped: 18

Q27 Over the course of your entire career, aproximately how many codes (cardiopulmonary resuscitations) have you attended?

Answered: 20 Skipped: 18

Q28 How old are you?

Answered: 20 Skipped: 18

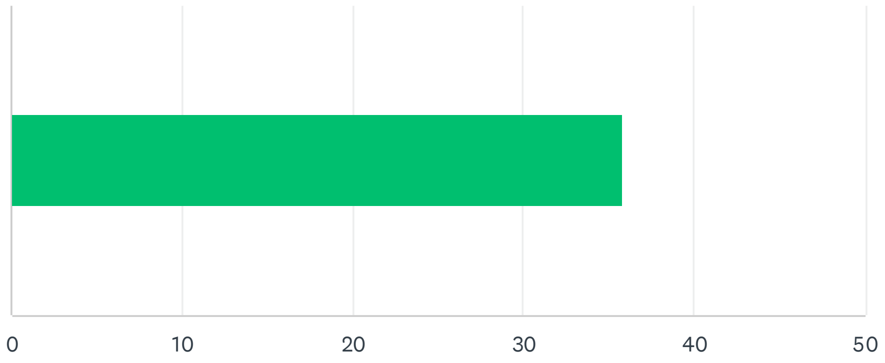

ANSWER CHOICES	AVERAGE NUMBER	TOTAL NUMBER	RESPONSES
	36	717	20
Total Respondents: 20			

Q29 What gender do you identify as?

Answered: 20   Skipped: 18

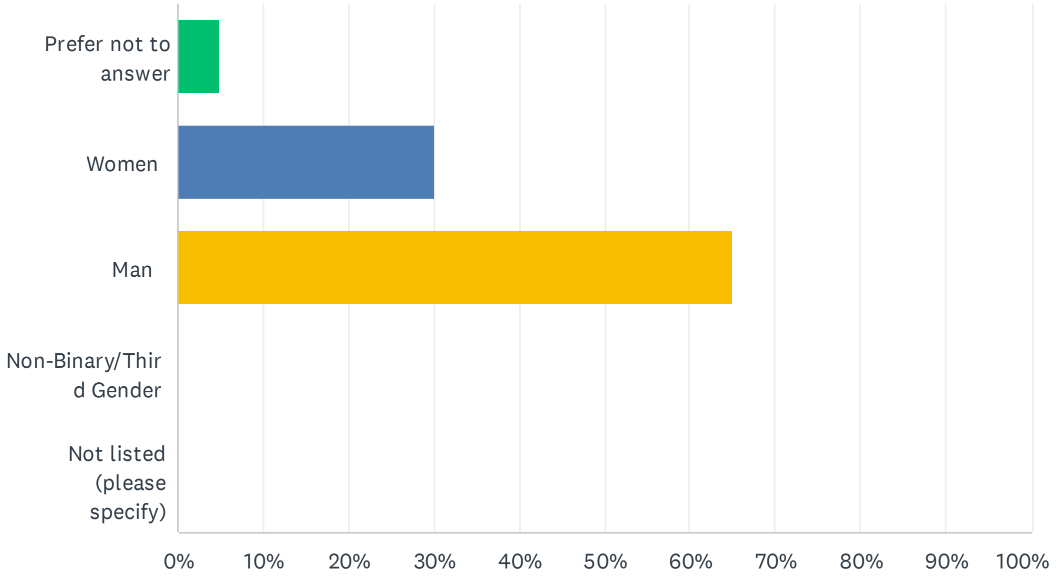

ANSWER CHOICES	RESPONSES	
Prefer not to answer	5.00%	1
Women	30.00%	6
Man	65.00%	13
Non-Binary/Third Gender	0.00%	0
Not listed (please specify)	0.00%	0
TOTAL		20

Q30 To examine the potential influence of sexual dimorphism (physiological differences in form between individuals of different sex such as differences in the average width of the palm between sexes), do you have lived experience as a trans person (meaning your gender identity does not align with your gender assigned at birth)?

Answered: 20 Skipped: 18

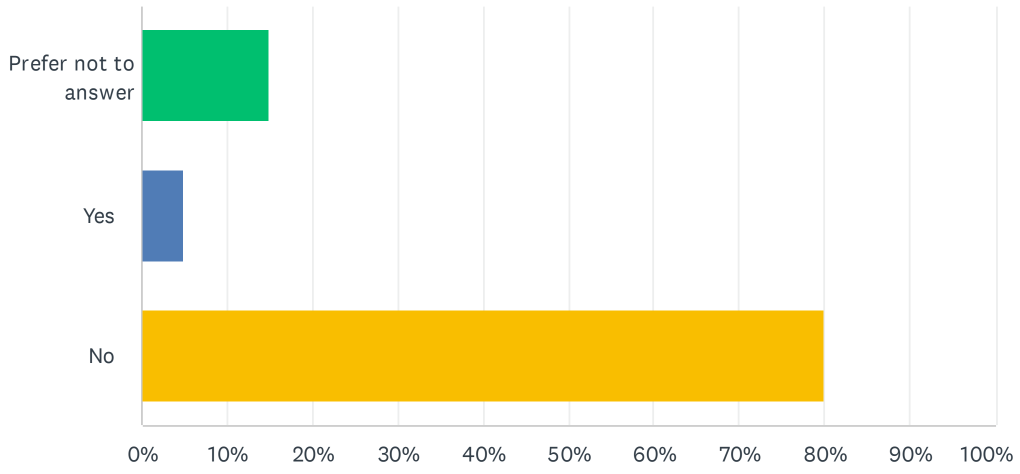

ANSWER CHOICES	RESPONSES	
Prefer not to answer	15.00%	3
Yes	5.00%	1
No	80.00%	16
TOTAL		20

Q31 Do you identify as a person currently living with a physical disability?

Answered: 20 Skipped: 18

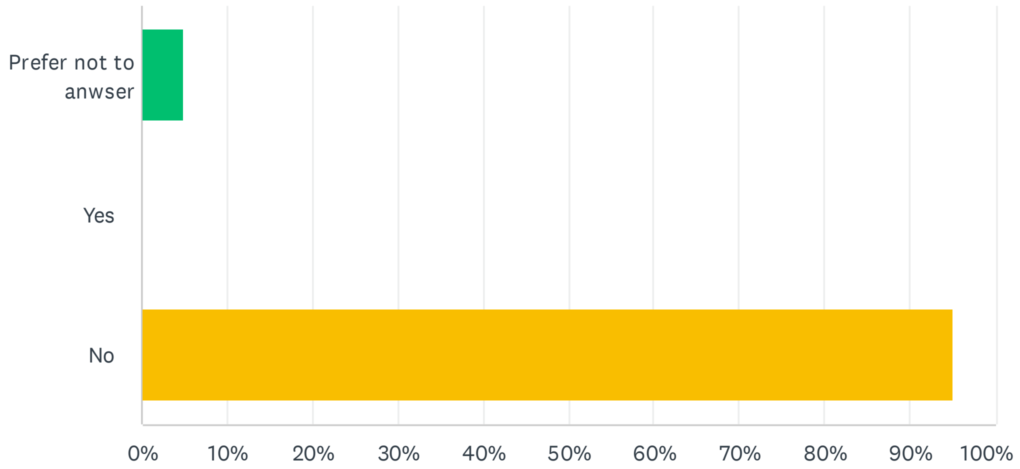

ANSWER CHOICES	RESPONSES	
Prefer not to anwser	5.00%	1
Yes	0.00%	0
No	95.00%	19
TOTAL		20

Q32 What is your cultural background? Choose all that apply.

Answered: 20   Skipped: 18

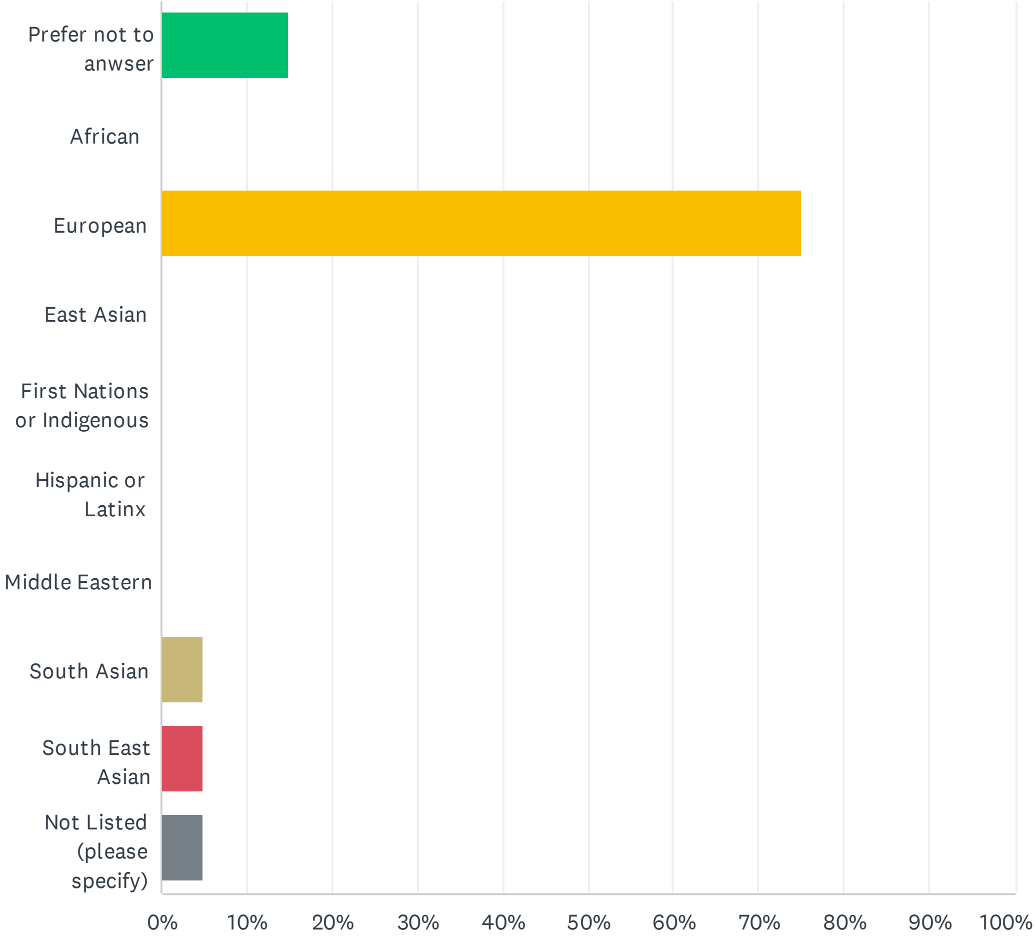

ANSWER CHOICES	RESPONSES	
Prefer not to anwser	15.00%	3
African	0.00%	0
European	75.00%	15
East Asian	0.00%	0
First Nations or Indigenous	0.00%	0
Hispanic or Latinx	0.00%	0
Middle Eastern	0.00%	0
South Asian	5.00%	1
South East Asian	5.00%	1
Not Listed (please specify)	5.00%	1
Total Respondents: 20		

Q33 Please specify your First Nations or Indigenous cultural background

Answered: 0 Skipped: 38

Q34 Do you have any additional comments you would like to share regarding your experience performing TEE-guided chest compressions before we move to the next part of the survey?

Answered: 9 Skipped: 29

Q35 If you are interested in participating in a follow-up interview, please provide you name and email address:

Answered: 3 Skipped: 35

ANSWER CHOICES	RESPONSES	
Name	100.00%	3
Company	0.00%	0
Address	0.00%	0
Address 2	0.00%	0
City/Town	0.00%	0
State/Province	0.00%	0
ZIP/Postal Code	0.00%	0
Country	0.00%	0
Email Address	100.00%	3
Phone Number	0.00%	0

Q1 Veuillez indiquer dans quelle province ou quel territoire vous résidezPlease indicate which province or territory you reside in

Answered: 14 Skipped: 0

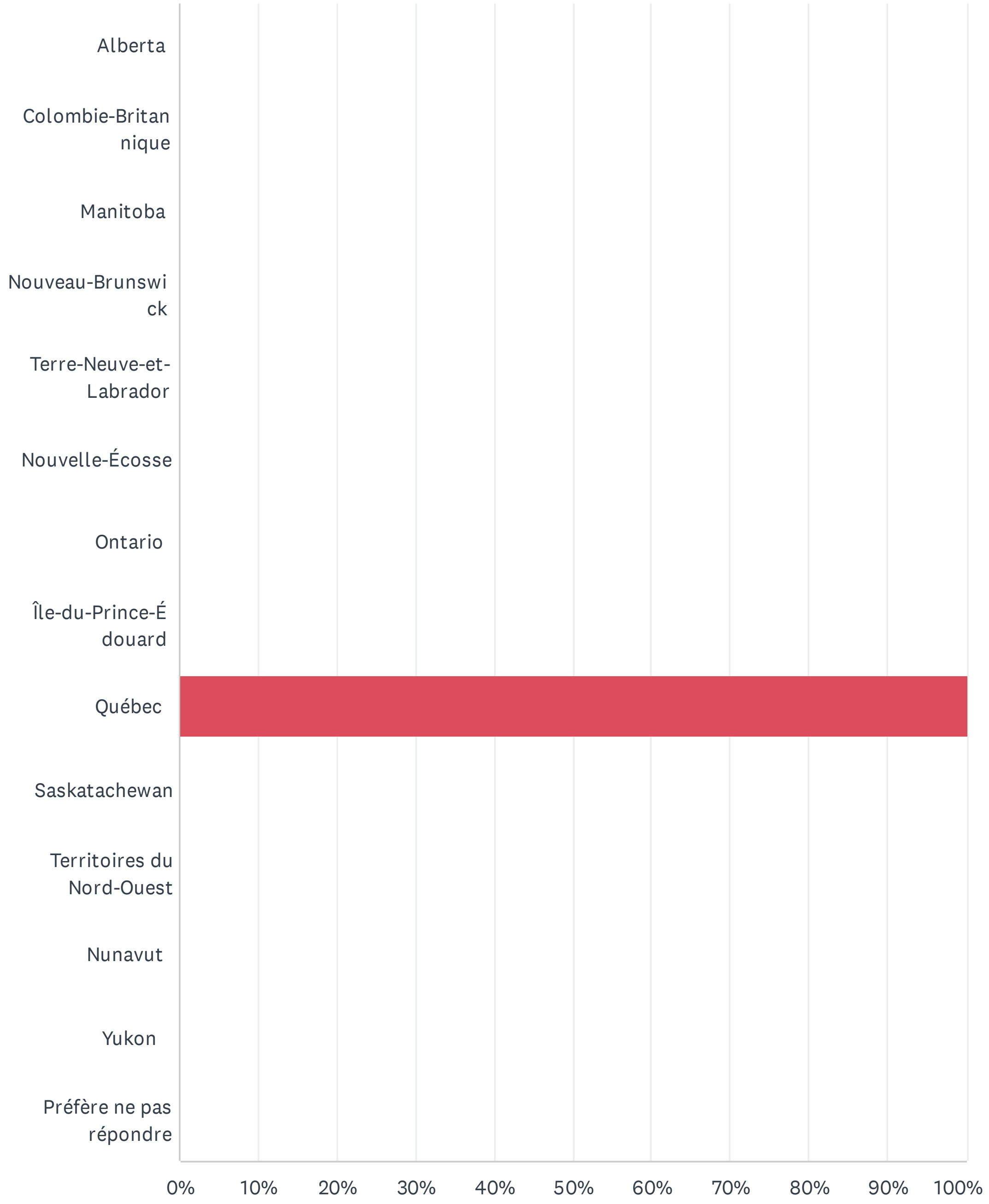

ANSWER CHOICES	RESPONSES	
Alberta	0.00%	0
Colombie-Britannique	0.00%	0
Manitoba	0.00%	0
Nouveau-Brunswick	0.00%	0
Terre-Neuve-et-Labrador	0.00%	0
Nouvelle-Écosse	0.00%	0
Ontario	0.00%	0
Île-du-Prince-Édouard	0.00%	0
Québec	100.00%	14
Saskatachewan	0.00%	0
Territoires du Nord-Ouest	0.00%	0
Nunavut	0.00%	0
Yukon	0.00%	0
Préfère ne pas répondre	0.00%	0
TOTAL		14

Q2 Facultatif : Dans quelle ville travaillez-vous ?Optional: What city do you work in?

Answered: 11 Skipped: 3

Q3 Avez-vous assisté à un code bleu (réanimation chez un patient en arrêt cardiaque) où un praticien a utilisé l'échocardiographie transœsophagienne (ETO) ? Si la réponse est non, nous vous remercions d'avoir prêté attention à ce sondage et nous vous demandons de cesser d'y répondre.Have you attended a code blue (cardiac arrest resuscitation) where a practitioner used trasesophageal echocardiography (TEE)? If not, we ask that you discontinue the survey at this time and thank you for your consideration.

Answered: 13 Skipped: 1

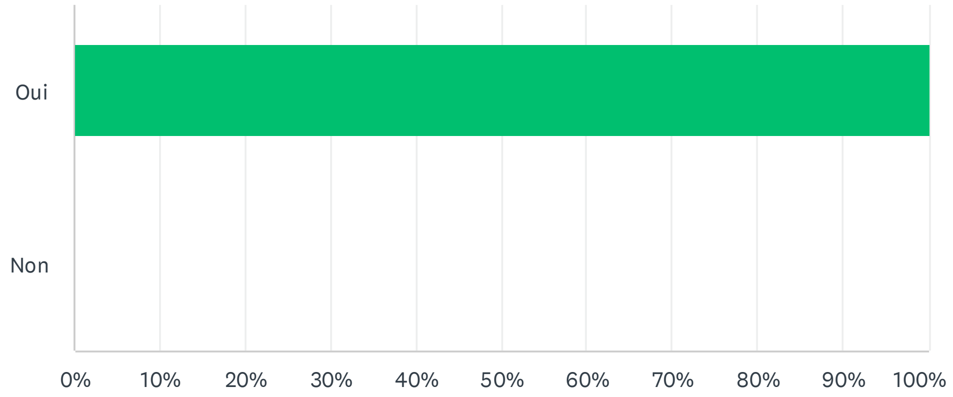

ANSWER CHOICES	RESPONSES	
Oui	100.00%	13
Non	0.00%	0
TOTAL		13

Q4 Avez-vous déjà effectué des compressions thoraciques sous la guidance de l'échocardiographie transœsophagienne ?Have you performed chest compressions under guidance from transesophageal echocardiography?

Answered: 13 Skipped: 1

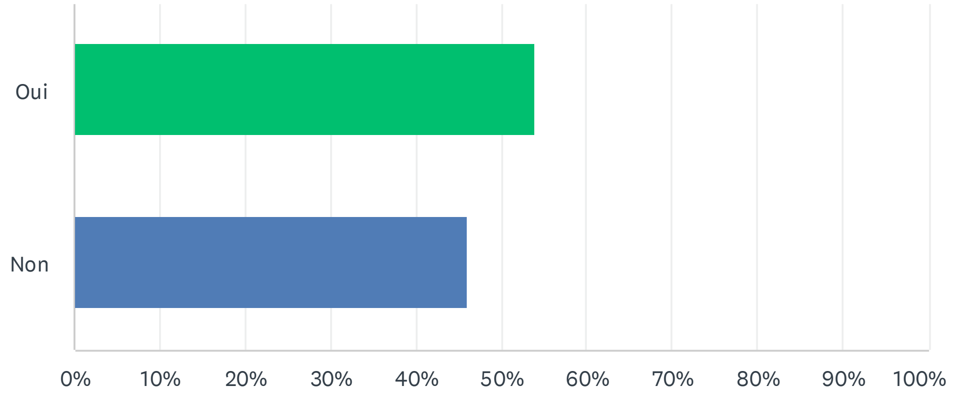

ANSWER CHOICES	RESPONSES	
Oui	53.85%	7
Non	46.15%	6
TOTAL		13

Q5 Avant l'utilisation de l'échocardiographie transœsophagienne (ETO) pour guider le massage, est-ce que les compressions thoraciques étaient effectuées sur la moitié inférieure du sternum/au centre de la poitrine, comme indiqué dans l'image ci-dessous (case F) ?Before TEE is being used to guide compression, were chest compressions performed on the lower half of the sternum/centre of the chest as shown by the image below (box F)

Answered: 8 Skipped: 6

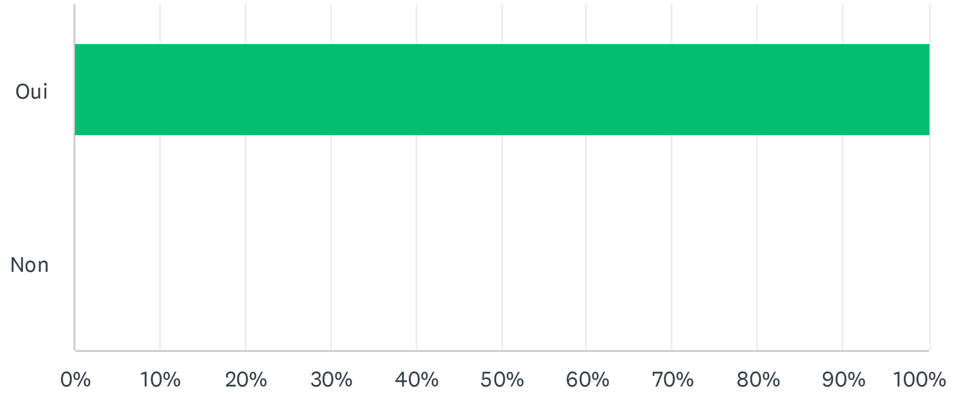

ANSWER CHOICES	RESPONSES	
Oui	100.00%	8
Non	0.00%	0
TOTAL		8

Q6 À quelle fréquence l'utilisation de l'échocardiographie transœsophagienne (ETO) pendant un arrêt cardiaque entraîne-t-elle des compressions thoraciques effectuées à un endroit différent sur le thorax? How often does the use of TEE during cardiac arrest result in compressions being performed on a different part of the chest?

Answered: 8 Skipped: 6

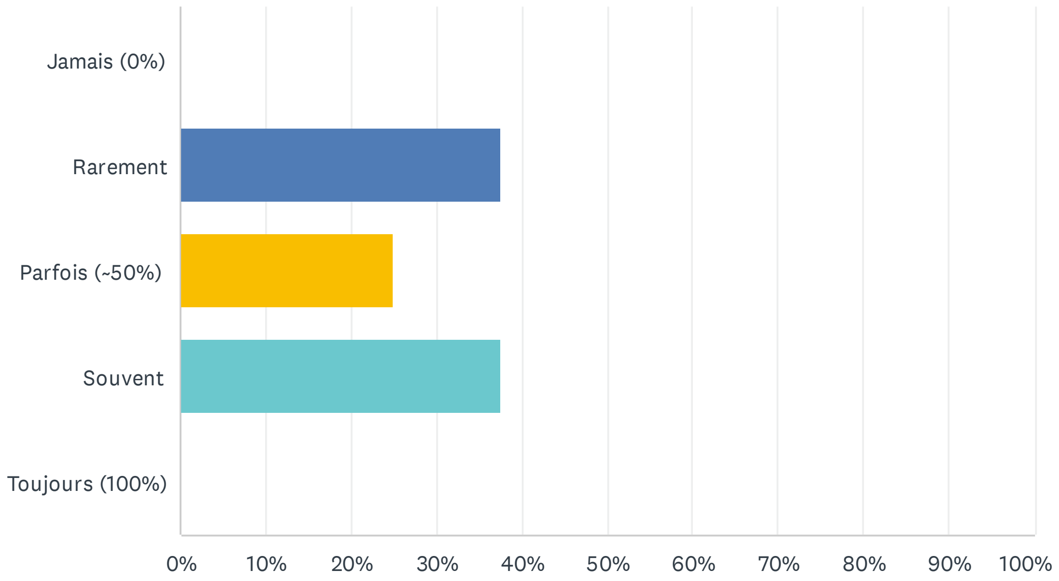

ANSWER CHOICES	RESPONSES	
Jamais (0%)	0.00%	0
Rarement	37.50%	3
Parfois (~50%)	25.00%	2
Souvent	37.50%	3
Toujours (100%)	0.00%	0
TOTAL		8

Q7 Lorsque l'échocardiographie transœsophagienne (ETO) est utilisée, est-ce qu'un membre de l'équipe vous demande généralement de changer l'endroit où vous effectuez les compressions thoraciques ?When TEE is in use, are you typically instructed by a member of the team to move where you are doing chest compressions?

Answered: 7 Skipped: 7

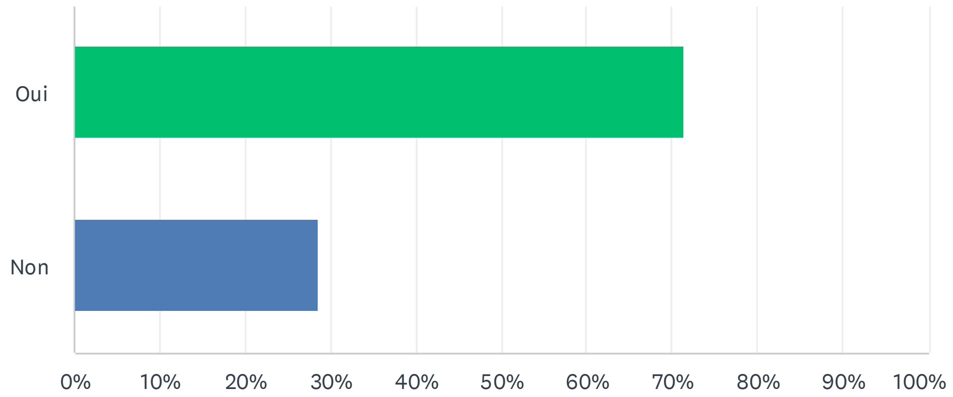

ANSWER CHOICES	RESPONSES	
Oui	71.43%	5
Non	28.57%	2
TOTAL		7

Q8 Si vous vous êtes généralement déplacés vers une autre zone que celle au centre du thorax, vers laquelle vous êtes-vous déplacés (cochez toutes les réponses qui s'appliquent) ?If you typically moved away from the center of the chest, where did you move to (check all that apply)?

Answered: 7 Skipped: 7

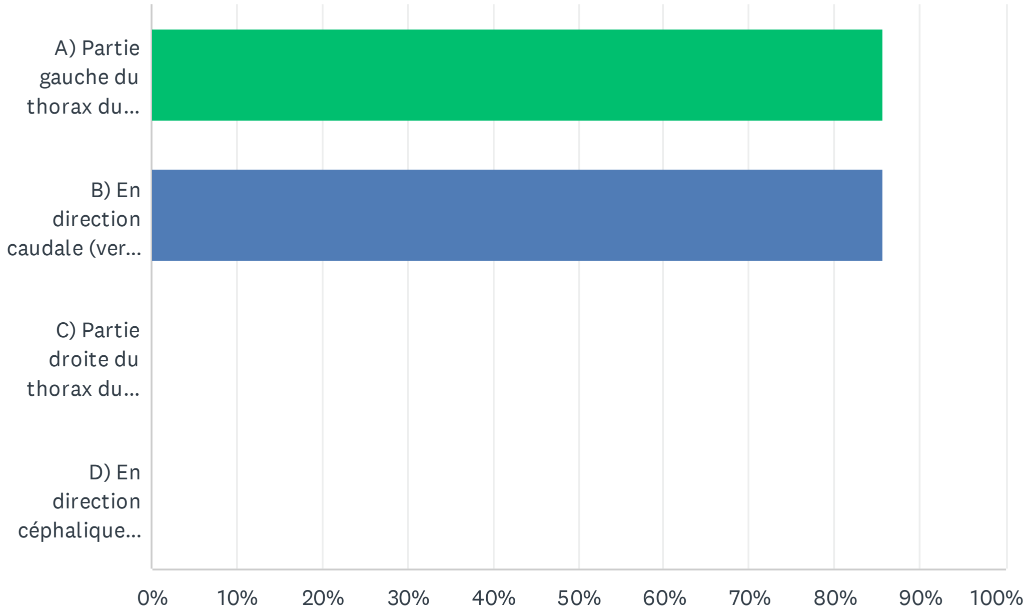

ANSWER CHOICES	RESPONSES	
A) Partie gauche du thorax du patient (vers le mamelon gauche)	85.71%	6
B) En direction caudale (vers les jambes)	85.71%	6
C) Partie droite du thorax du patient (vers le mamelon droit)	0.00%	0
D) En direction céphalique (vers la tête)	0.00%	0
Total Respondents: 7		

Q9 En général, vous a-t-on donné des instructions verbales pour vous déplacer vers une nouvelle zone de compression ?Typically, were you given verbal instructions to move to a new area of compression?

Answered: 7 Skipped: 7

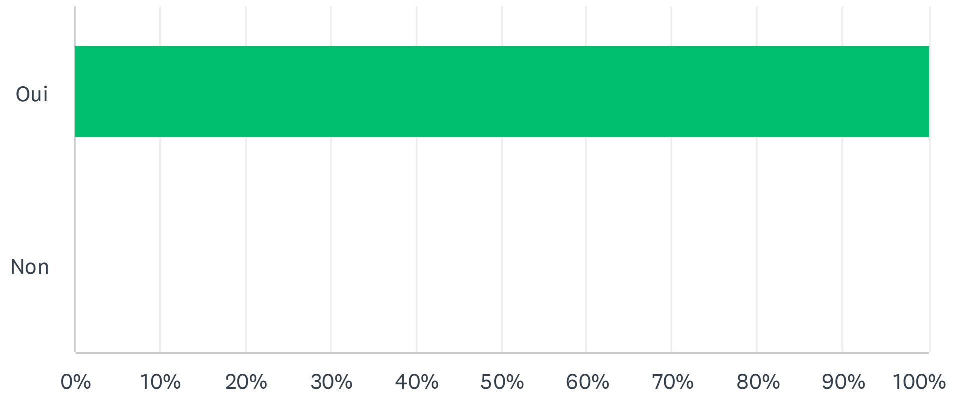

ANSWER CHOICES	RESPONSES	
Oui	100.00%	7
Non	0.00%	0
TOTAL		7

Q10 Généralement, avez-vous reçu de la rétroaction ou des instructions verbales de l'équipe pour vous guider lorsque vous aviez atteint la zone idéale de compression?Typically, were you provided verbal feedback/instructions from the team to guide you when you were at the correct area of compression?

Answered: 7 Skipped: 7

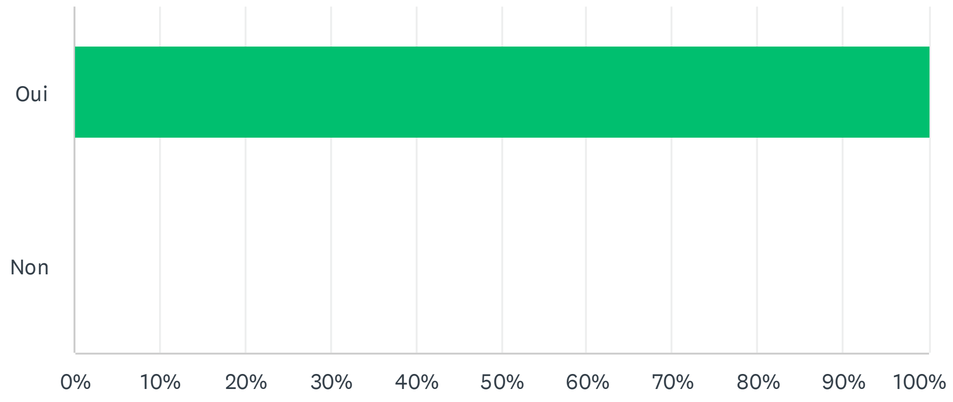

ANSWER CHOICES	RESPONSES	
Oui	100.00%	7
Non	0.00%	0
TOTAL		7

Q11 Généralement, vous a-t-on donné de la rétroaction visuelle via l'écran d'échocardiographie pour guider vos compressions thoraciques ?Typically, were you provided visual feedback from the echocardiography screen to guide your compressions?

Answered: 7 Skipped: 7

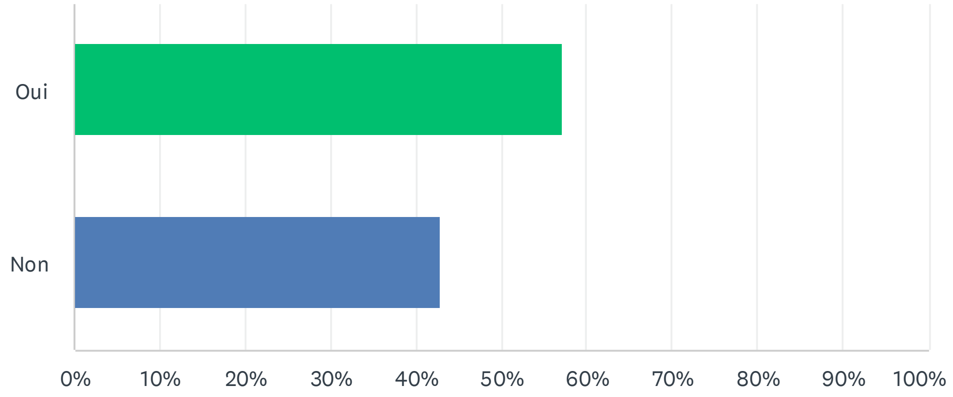

ANSWER CHOICES	RESPONSES	
Oui	57.14%	4
Non	42.86%	3
TOTAL		7

Q12 En utilisant la lettre correspondante à la zone, dans quelle région commenciez-vous généralement la RCR avant l'utilisation de l'échocardiographie transœsophagienne (ETO) ?Using the corresponding zone letter, what area did you typically start CPR in before TEE?

Answered: 7 Skipped: 7

Q13 En utilisant les lettres correspondant aux zones, veuillez indiquer, par ordre de fréquence (1 étant le plus fréquent), les 4 emplacements principaux où vous effectuez les compressions thoraciques après le début de l'échocardiographie transœsophagienne (ETO). Si vous utilisez moins de 4 emplacements, laissez les cases vides.Using the corresponding zone letters, please list in order of frequency (1 being most common) the top 4 locations you typically perform compressions after TEE is initiated. If you use less than 4 locations, leave the empty boxes blank.

Answered: 7 Skipped: 7

ANSWER CHOICES	RESPONSES	
1. (le plus fréquent)	100.00%	7
2.	85.71%	6
3.	42.86%	3
4.	28.57%	2

Q14 Avez-vous perçu une différence dans la compliance thoracique (par exemple, la flexion ou le rebondissement de la paroi thoracique)?Did you perceive a difference in chest compliance (examples include flex or recoil of the chest wall)?

Answered: 6   Skipped: 8

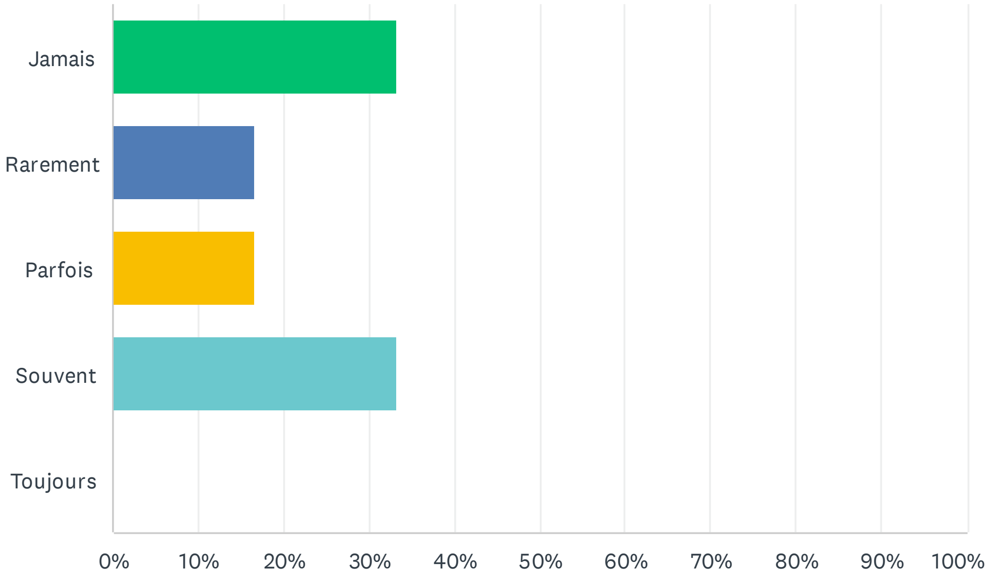

ANSWER CHOICES	RESPONSES	
Jamais	33.33%	2
Rarement	16.67%	1
Parfois	16.67%	1
Souvent	33.33%	2
Toujours	0.00%	0
TOTAL		6

Q15 Y avait-il une différence dans la flexion de la poitrine (résistance à la compression de la poitrine pendant les compressions)?Was there a difference in chest flexion (resistance to the chest being compressed during compressions)?

Answered: 6 Skipped: 8

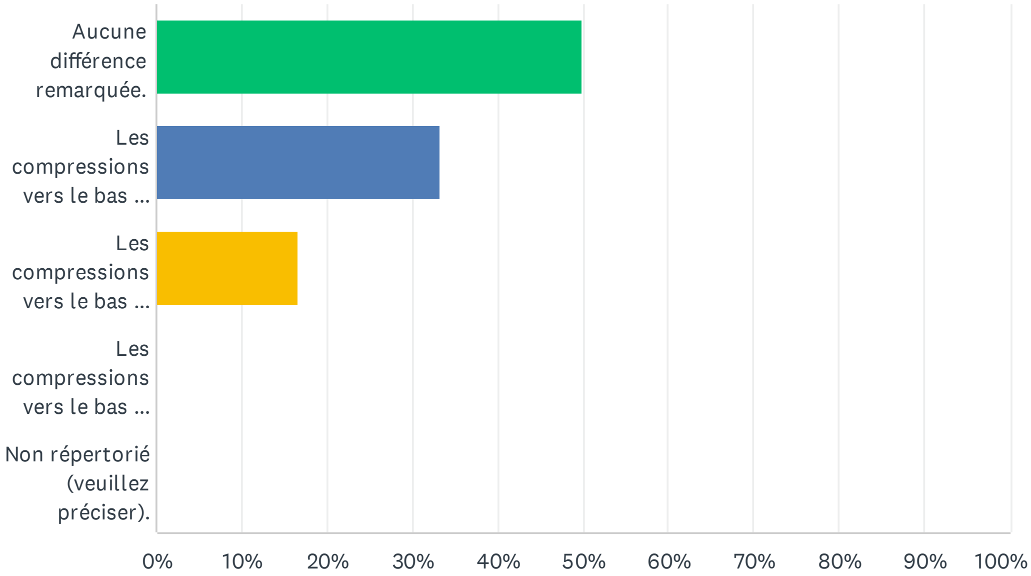

ANSWER CHOICES	RESPONSES	
Aucune différence remarquée.	50.00%	3
Les compressions vers le bas de la poitrine étaient plus difficiles.	33.33%	2
Les compressions vers le bas de la poitrine étaient plus faciles.	16.67%	1
Les compressions vers le bas de la poitrine étaient les mêmes.	0.00%	0
Non répertorié (veuillez préciser).	0.00%	0
TOTAL		6

Q16 Y avait-il une différence dans le retour à la forme non-comprimée/anatomique de la poitrine (rebondissement)?Was there a difference in chest recoil (degree to which the chest returns to non-compressed/anatomic shape)?

Answered: 6 Skipped: 8

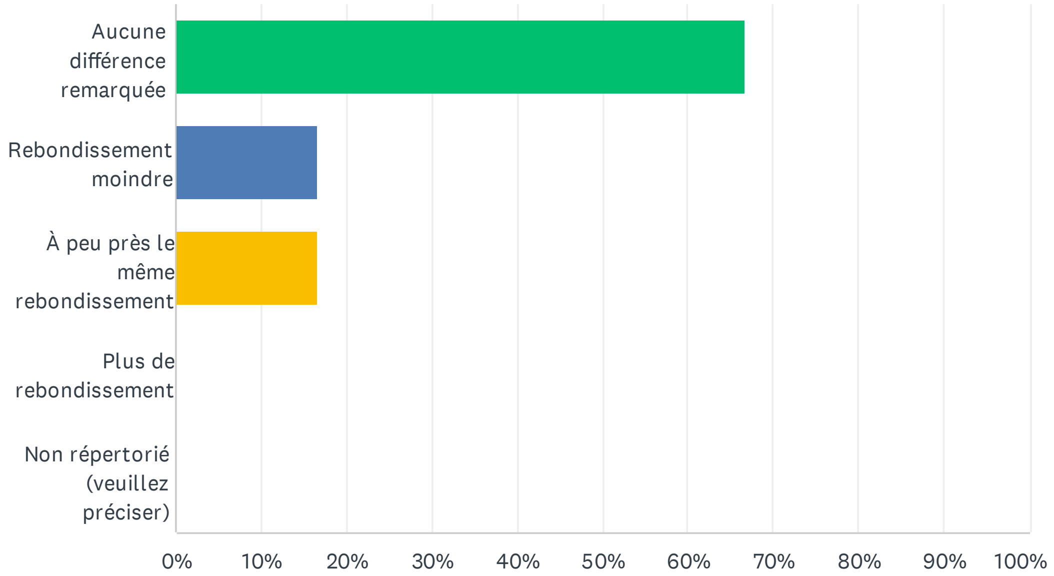

ANSWER CHOICES	RESPONSES	
Aucune différence remarquée	66.67%	4
Rebondissement moindre	16.67%	1
À peu près le même rebondissement	16.67%	1
Plus de rebondissement	0.00%	0
Non répertorié (veuillez préciser)	0.00%	0
Total Respondents: 6		

Q17 Est-il généralement difficile de maintenir l'emplacement identifié des compressions thoraciques guidées par ETO entre les vérifications de pouls/rythme?Once identified, is the TEE-guided location of compression typically challenging to maintain between pulse/rhythm checks?

Answered: 6 Skipped: 8

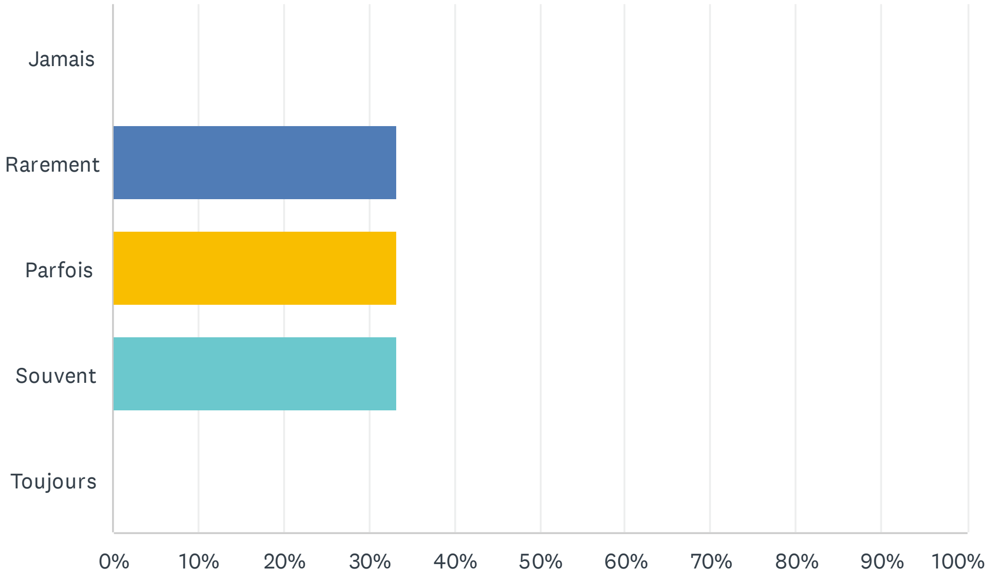

ANSWER CHOICES	RESPONSES	
Jamais	0.00%	0
Rarement	33.33%	2
Parfois	33.33%	2
Souvent	33.33%	2
Toujours	0.00%	0
TOTAL		6

Q18 Lors des compressions thoraciques, êtes-vous généralement en mesure de maintenir les compressions sur l'emplacement identifié par l'échocardiographie transœsophagienne (ETO)?During chest compressions, are you typically able to maintain compressions over the TEE-guided location?

Answered: 6 Skipped: 8

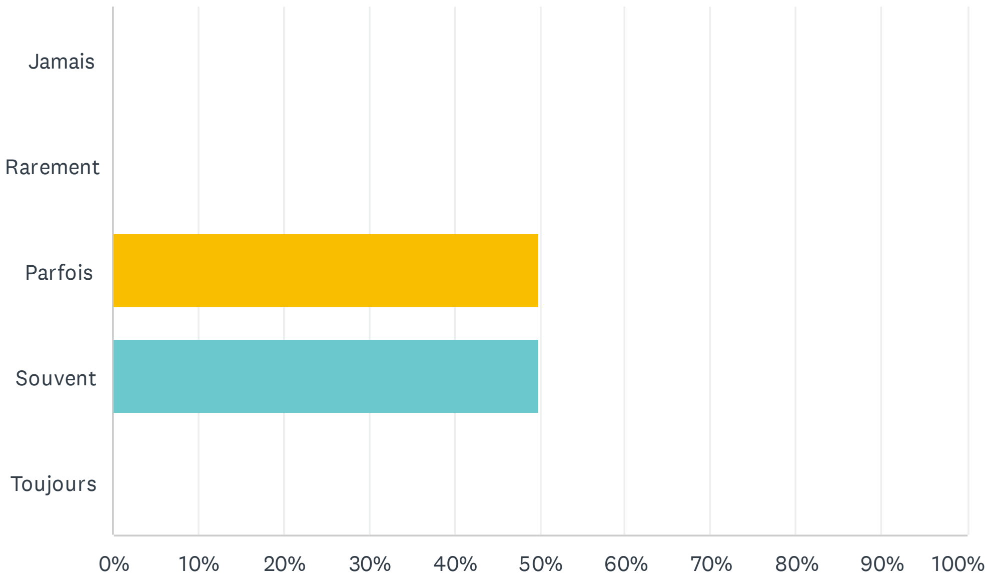

ANSWER CHOICES	RESPONSES	
Jamais	0.00%	0
Rarement	0.00%	0
Parfois	50.00%	3
Souvent	50.00%	3
Toujours	0.00%	0
TOTAL		6

Q19 Quels étaient, en général, les défis qui ont empêché d'effectuer les compressions thoraciques dans la zone thoracique identifiée ? Par exemple, moins de structure, absence de repères anatomiques, ergonomie, etc.What typically were the challenges that lead to not being able to perform compressions in the adjusted area of the chest?For example, less structure, no land mark, ergonomics, etc.

Answered: 6 Skipped: 8

Q20 Quel est votre perception de l'effort nécessaire pour effectuer des compressions thoraciques en dehors de la moitié inférieure du sternum/du centre de la poitrine ?Rate your perceived effort of performing chest compressions off the lower half of sternum/centre of chest?

Answered: 6 Skipped: 8

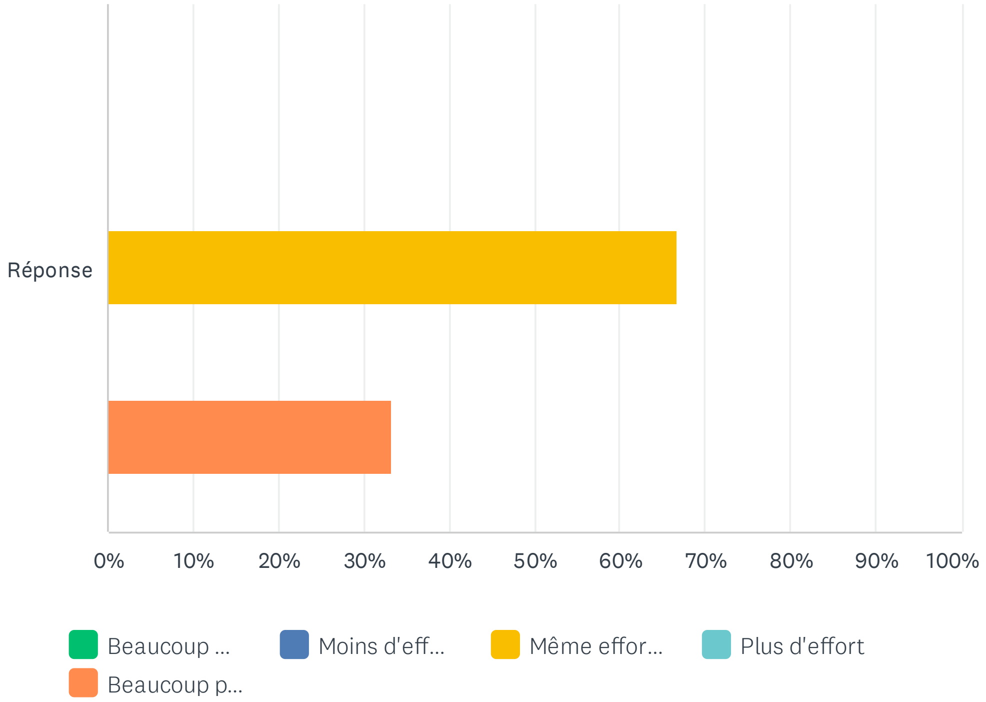

	BEAUCOUP MOINS D'EFFORT	MOINS D'EFFORT	MÊME EFFORT (NEUTRE)	PLUS D'EFFORT	BEAUCOUP PLUS D'EFFORT	TOTAL
Réponse	0.00%<br>0	0.00%<br>0	66.67%<br>4	0.00%<br>0	33.33%<br>2	6

Q21 Vous montre-t-on les images provenant de la machine d'échocardiographie transœsophagienne (ETO) pour vous aider à guider la qualité de vos compressions thoraciques (fréquence, profondeur, emplacement, etc.)?Are you ever shown images from the TEE machine to help you guide your compression quality (rate, depth, location, etc.)?

Answered: 6 Skipped: 8

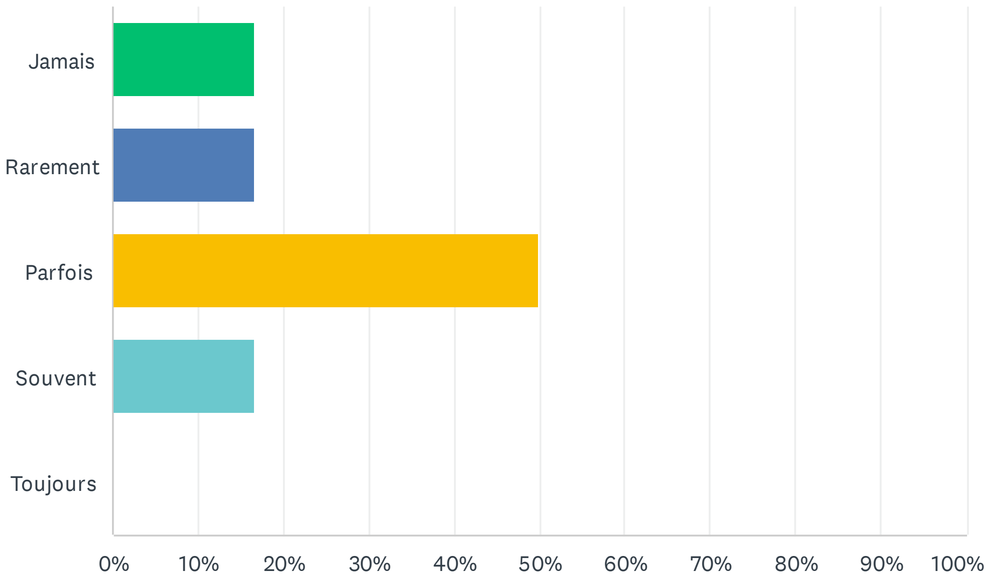

ANSWER CHOICES	RESPONSES	
Jamais	16.67%	1
Rarement	16.67%	1
Parfois	50.00%	3
Souvent	16.67%	1
Toujours	0.00%	0
TOTAL		6

Q22 Comment l'utilisation de l'ETO pendant la réanimation a-t-elle influencé la performance globale de l'équipe?How did having TEE in the resuscitation impact the overall performance of the team?

Answered: 10   Skipped: 4

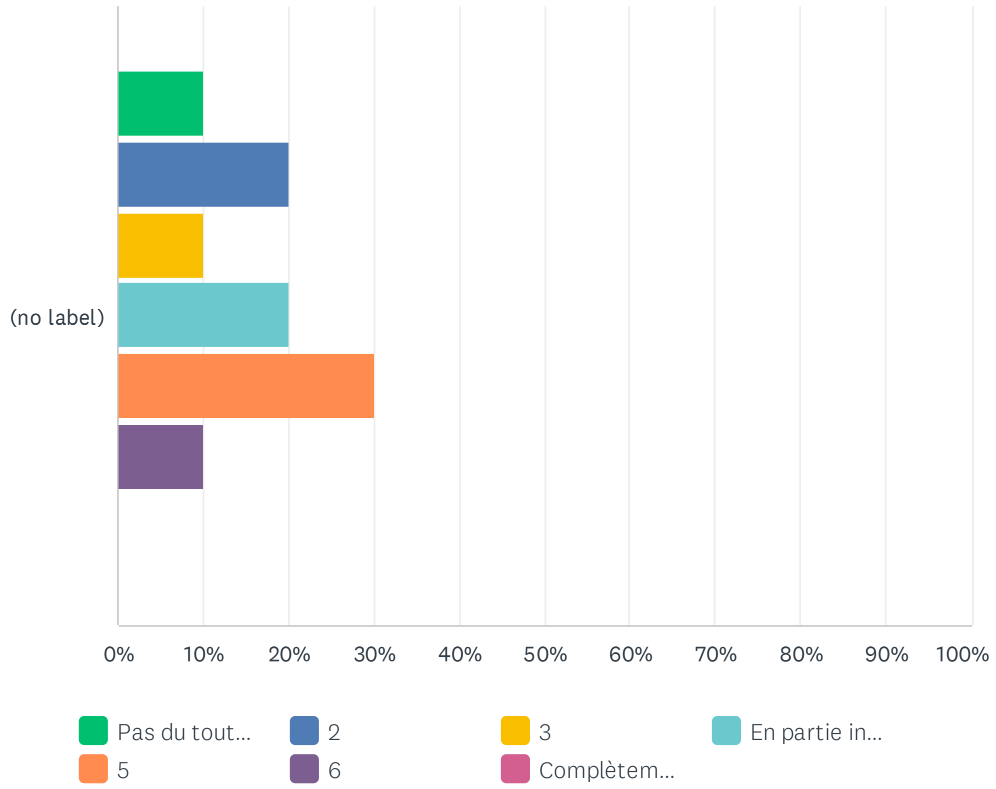

	PAS DU TOUT INFLUENCÉE1	2	3	EN PARTIE INFLUENCÉE4	5	6	COMPLÈTEMENT CHANGÉE7	TOTAL	WEIGHTED AVERAGE
(no label)	10.00%<br>1	20.00%<br>2	10.00%<br>1	20.00%<br>2	30.00%<br>3	10.00%<br>1	0.00%<br>0	10	1.00

Q23 Comment l'utilisation de l'ETO a-t-elle influencé le leadership au sein de la réanimation?How did TEE impact the leadership within the resuscitation?

Answered: 9 Skipped: 5

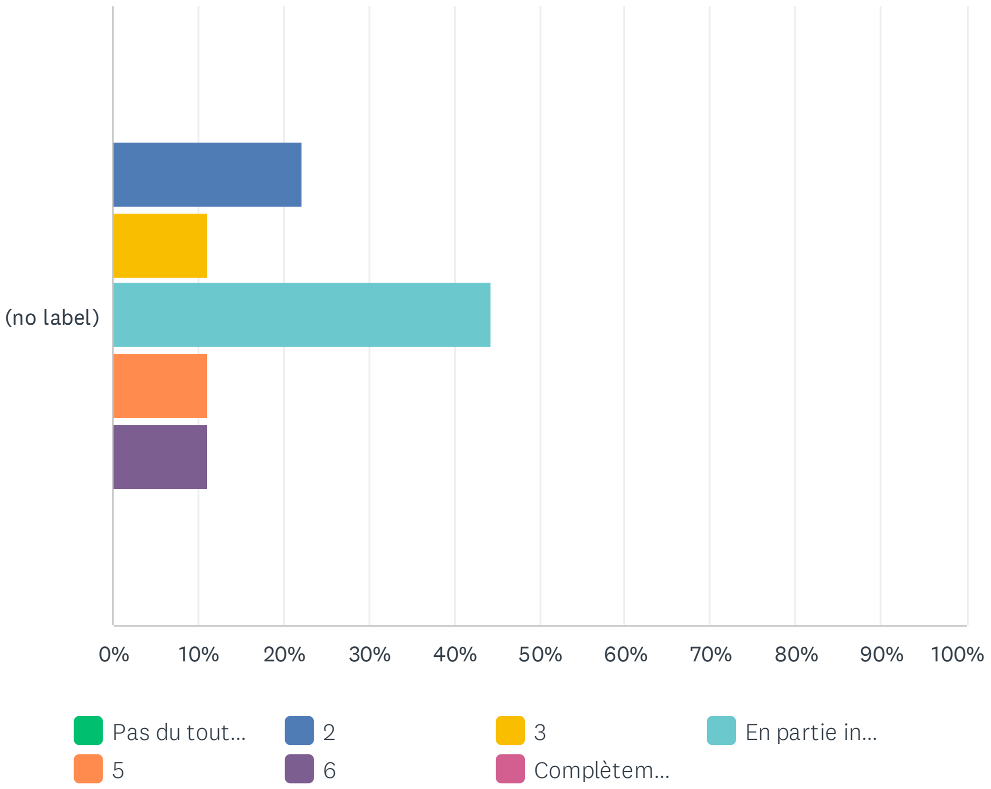

	PAS DU TOUT INFLUENCÉ1	2	3	EN PARTIE INFLUENCÉ4	5	6	COMPLÈTEMENT CHANGÉ7	TOTAL	WEIGHTED AVERAGE
(no label)	0.00%<br>0	22.22%<br>2	11.11%<br>1	44.44%<br>4	11.11%<br>1	11.11%<br>1	0.00%<br>0	9	1.00

Q24 Comment l'ETO a-t-elle influencé la résolution de problèmes pendant la réanimation?How did TEE impact problem solving during the resuscitation?

Answered: 9 Skipped: 5

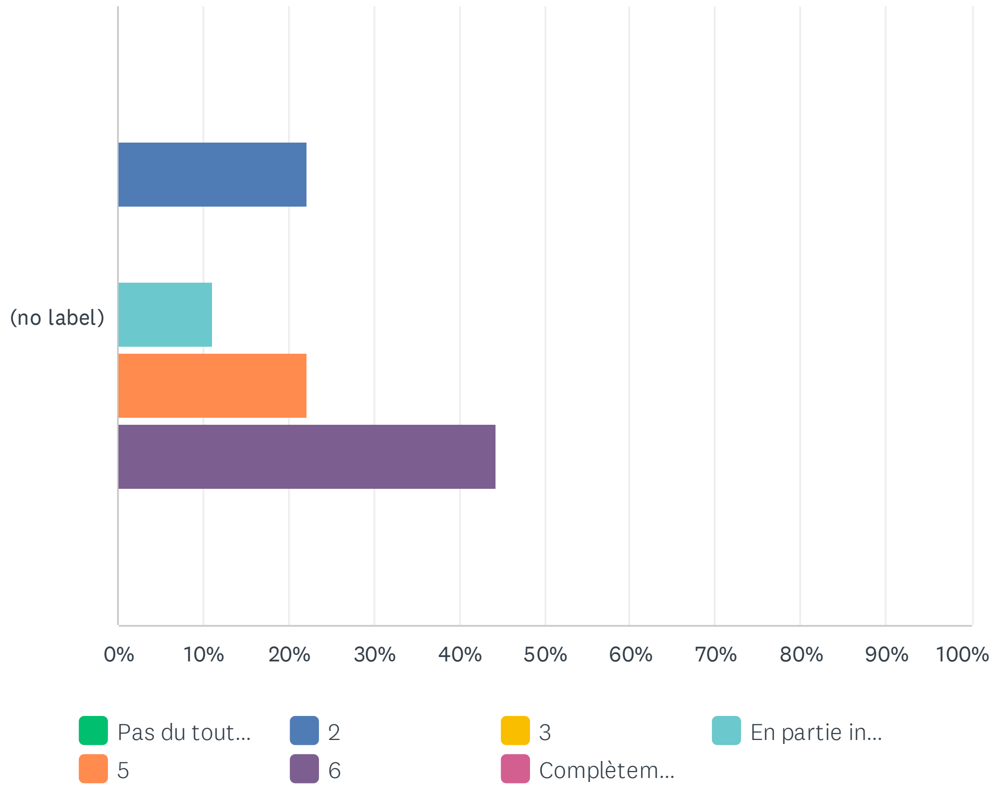

	PAS DU TOUT INFLUENCÉE1	2	3	EN PARTIE INFLUENCÉE4	5	6	COMPLÈTEMENT CHANGÉE7	TOTAL	WEIGHTED AVERAGE
(no label)	0.00%<br>0	22.22%<br>2	0.00%<br>0	11.11%<br>1	22.22%<br>2	44.44%<br>4	0.00%<br>0	9	1.00

Q25 Comment l'ETO a-t-elle influencé la prise de conscience et l'attention portées à d'autres aspects-clés de la réanimation tels que le contrôle du rythme, la vérification de pouls, les médicaments, etc,?How did the TEE impact situational awareness and attention to other key aspects of code blue like rhythm check, pulse check, meds, etc.?

Answered: 8 Skipped: 6

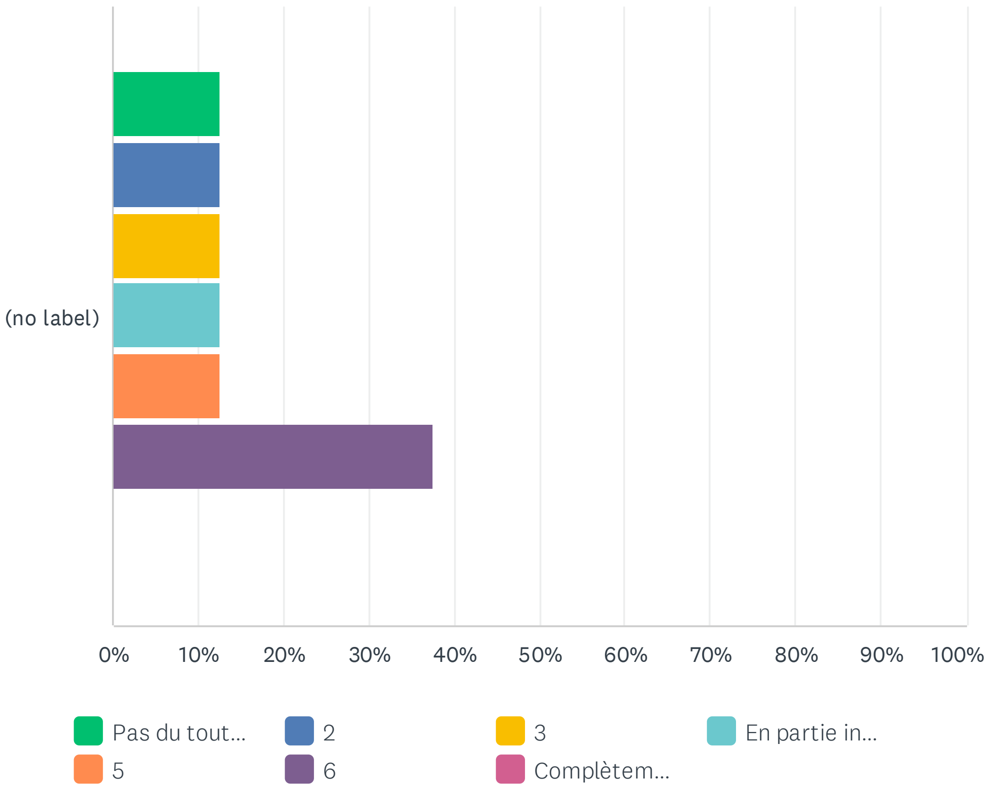

	PAS DU TOUT INFLUENCÉES1	2	3	EN PARTIE INFLUENCÉES4	5	6	COMPLÈTEMENT CHANGÉES7	TOTAL	WEIGHTE[illegible] AVERAGE
(no label)	12.50% 1	12.50% 1	12.50% 1	12.50% 1	12.50% 1	37.50% 3	0.00% 0	8	1.[illegible]

Q26 Comment l'ETO a-t-elle influencé l'utilisation des ressources?How did TEE impact resource utilization?

Answered: 8 Skipped: 6

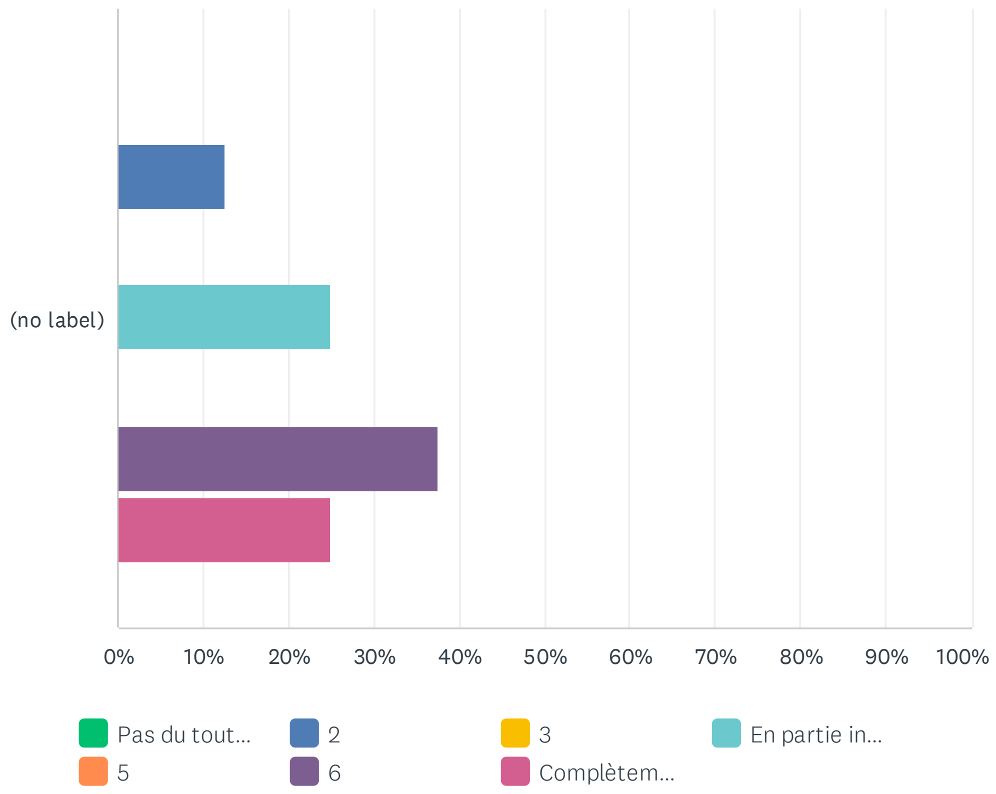

	PAS DU TOUT INFLUENCÉE1	2	3	EN PARTIE INFLUENCÉE4	5	6	COMPLÈTEMENT CHANGÉE7	TOTAL	WEIGHTED AVERAGE
(no label)	0.00% 0	12.50% 1	0.00% 0	25.00% 2	0.00% 0	37.50% 3	25.00% 2	8	1.00

Q27 Comment l'ETO a-t-elle influencé la communication dans la salle de réanimation?How did TEE impact communication within the room?

Answered: 8   Skipped: 6

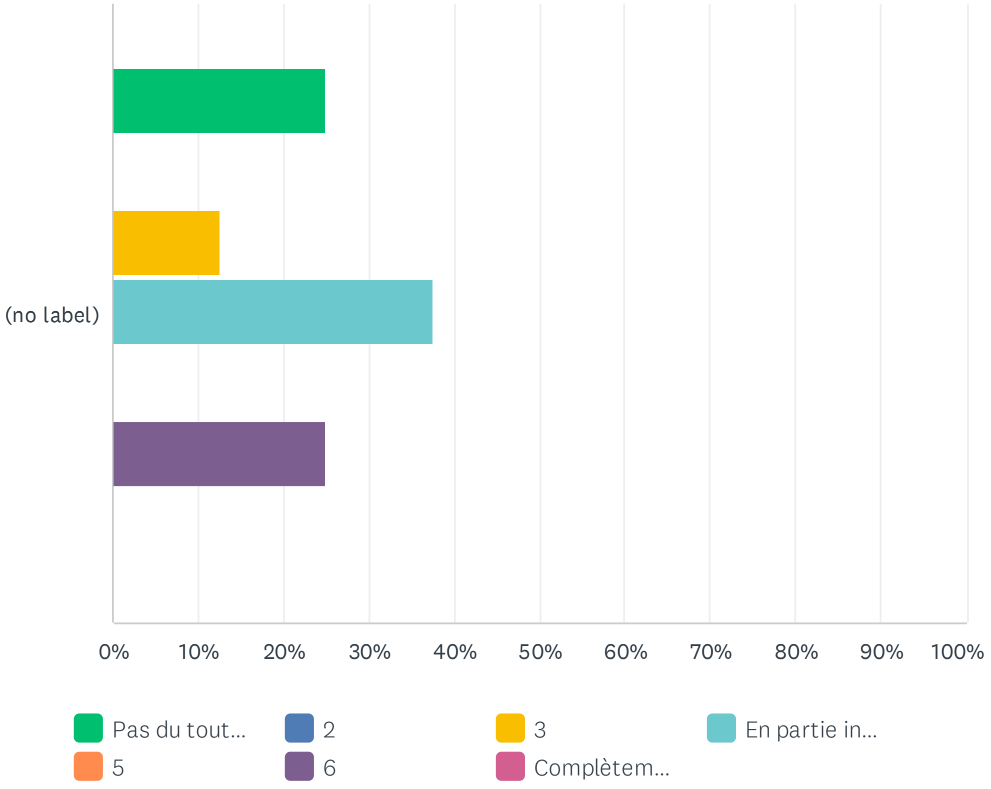

	PAS DU TOUT INFLUENCÉE1	2	3	EN PARTIE INFLUENCÉE4	5	6	COMPLÈTEMENT CHANGÉE7	TOTAL	WEIGHTED AVERAGE
(no label)	25.00%<br>2	0.00%<br>0	12.50%<br>1	37.50%<br>3	0.00%<br>0	25.00%<br>2	0.00%<br>0	8	1.00

Q28 Quel est le titre de votre emploi actuel au sein de l'équipe de réanimation ?What is your current job title in the context of performing resuscitation?

Answered: 8 Skipped: 6

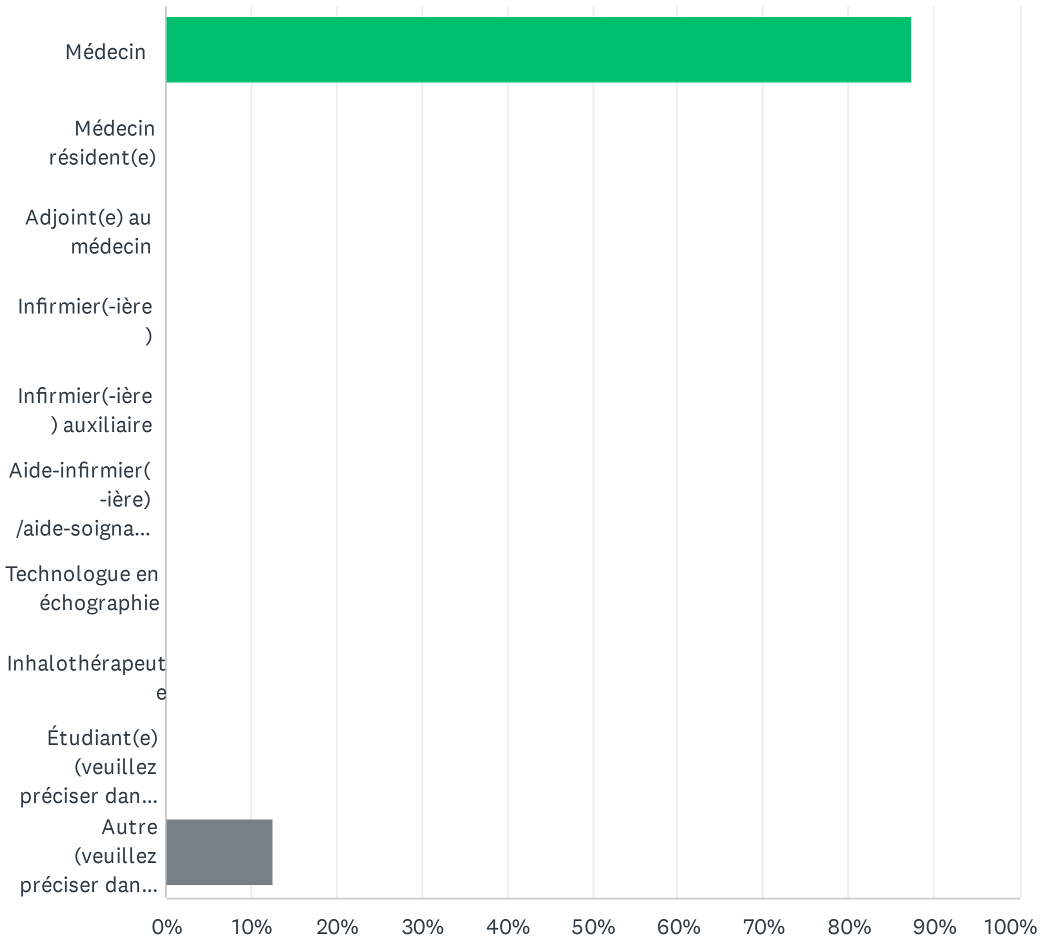

ANSWER CHOICES	RESPONSES	
Médecin	87.50%	7
Médecin résident(e)	0.00%	0
Adjoint(e) au médecin	0.00%	0
Infirmier(-ière)	0.00%	0
Infirmier(-ière) auxiliaire	0.00%	0
Aide-infirmier(-ière) /aide-soignant(e)/préposé(e) aux bénéficiaires	0.00%	0
Technologue en échographie	0.00%	0
Inhalothérapeute	0.00%	0
Étudiant(e) (veuillez préciser dans la boîte de texte)	0.00%	0
Autre (veuillez préciser dans la boîte de texte)	12.50%	1
TOTAL		8

Q29 Depuis combien d'années êtes-vous en pratique (nombre arrondi à l'année la plus proche)?How many years have you been in practice (round to the nearest year)?

Answered: 8   Skipped: 6

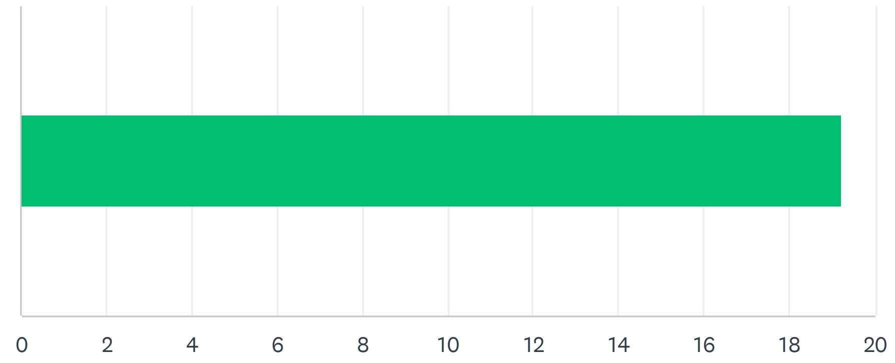

ANSWER CHOICES	AVERAGE NUMBER	TOTAL NUMBER	RESPONSES
	19	154	8
Total Respondents: 8			

Q30 Combien d'années d'expérience avez-vous en réanimation à votre poste actuel (nombre arrondi à l'année la plus proche)?How many years experience do you have performing resuscitation in your current role (round to nearest year)?

Answered: 8 Skipped: 6

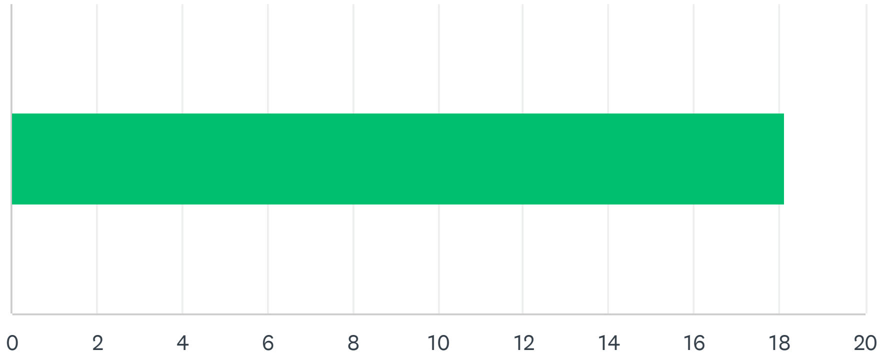

ANSWER CHOICES	AVERAGE NUMBER	TOTAL NUMBER	RESPONSES
	18	145	8
Total Respondents: 8			

Q31 Au cours d'une année, à combien (approximativement) de réanimations chez un patient en arrêt cardiaque (codes bleus) participez-vous?In an average year, approximately how many codes (cardiopulmonary resuscitations) do you attend?

Answered: 8   Skipped: 6

Q32 Au cours de votre carrière, à combien (approximativement) de réanimations chez un patient en arrêt cardiaque (codes bleus) avez-vous participé? Over the course of your entire career, approximately how many codes (cardiopulmonary resuscitations) have you attended?

Answered: 8 Skipped: 6

Q33 Quel âge avez-vous ? How old are you?

Answered: 8 Skipped: 6

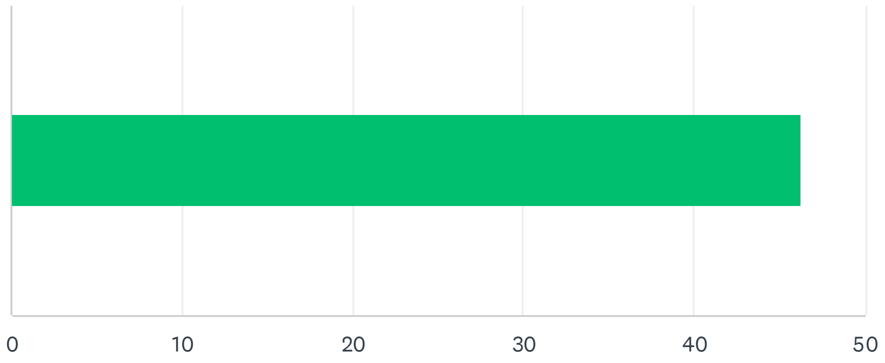

ANSWER CHOICES	AVERAGE NUMBER	TOTAL NUMBER	RESPONSES
	46	370	8
Total Respondents: 8			

Q34 À quel genre vous identifiez-vous?What gender do you identify as?

Answered: 8 Skipped: 6

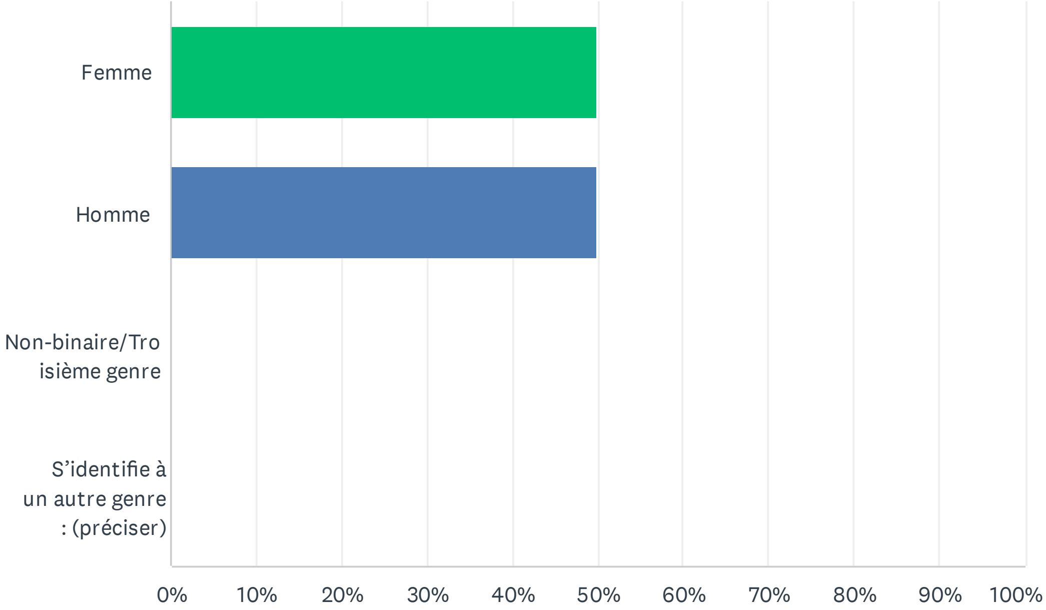

ANSWER CHOICES	RESPONSES	
Femme	50.00%	4
Homme	50.00%	4
Non-binaire/Troisième genre	0.00%	0
S'identifie à un autre genre : (préciser)	0.00%	0
TOTAL		8

Q35 Pour étudier l'influence potentielle du dimorphisme sexuel (différences physiologiques dues à la taille entre les individus de sexe différent comme la différence de largeur moyenne de la paume de la main entre les sexes), vous identifiez-vous comme personne trans (ce qui signifie que votre identité de genre ne correspond pas à votre sexe assigné à la naissance) ?To examine the potential influence of sexual dimorphism (physiological differences in form between individuals of different sex such as differences in the average width of the palm between sexes), do you have lived experience as a trans person (meaning your gender identity does not align with your gender assigned at birth)?

Answered: 8 Skipped: 6

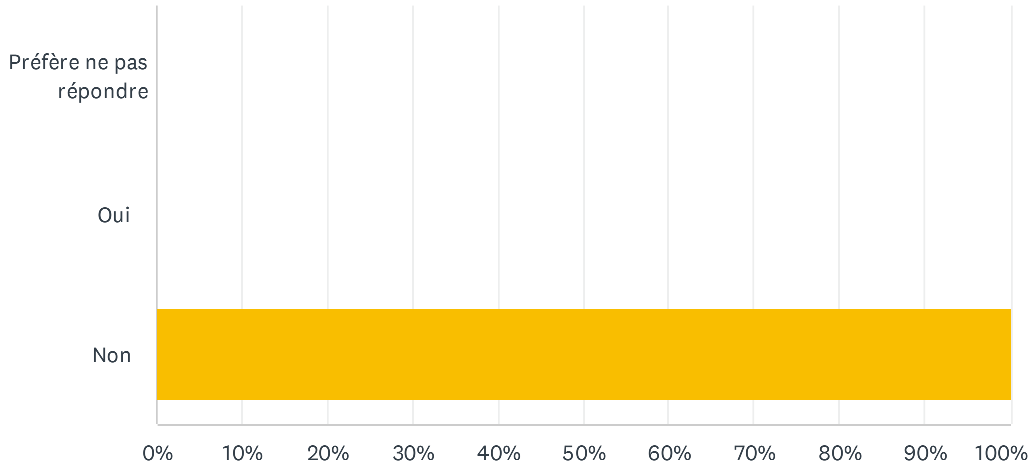

ANSWER CHOICES	RESPONSES	
Préfère ne pas répondre	0.00%	0
Oui	0.00%	0
Non	100.00%	8
TOTAL		8

Q36 Vous identifiez-vous en tant que personne vivant avec un handicap physique ?Do you identify as a person currently living with a physical disability?

Answered: 8   Skipped: 6

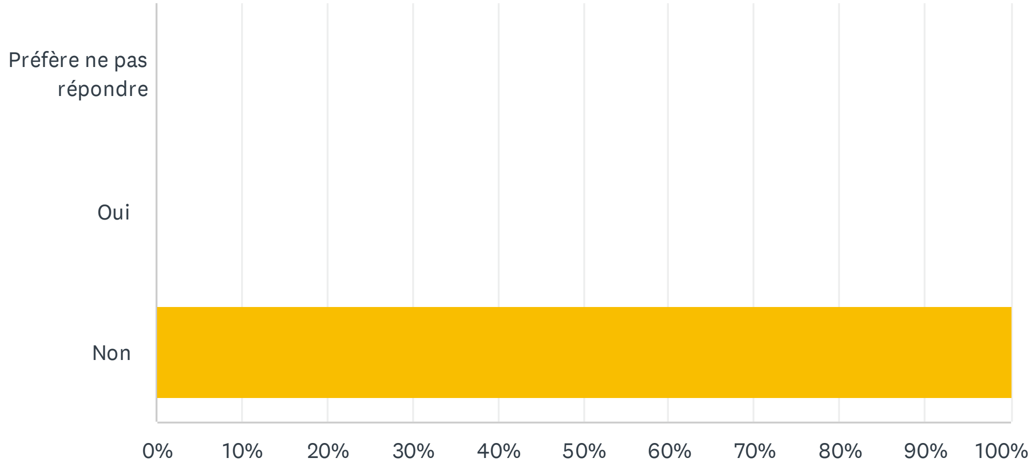

ANSWER CHOICES	RESPONSES	
Préfère ne pas répondre	0.00%	0
Oui	0.00%	0
Non	100.00%	8
TOTAL		8

Q37 Quelle est votre origine culturelle ? Sélectionnez toutes les réponses qui s'appliquent :What is your cultural background? Choose all that apply.

Answered: 8 Skipped: 6

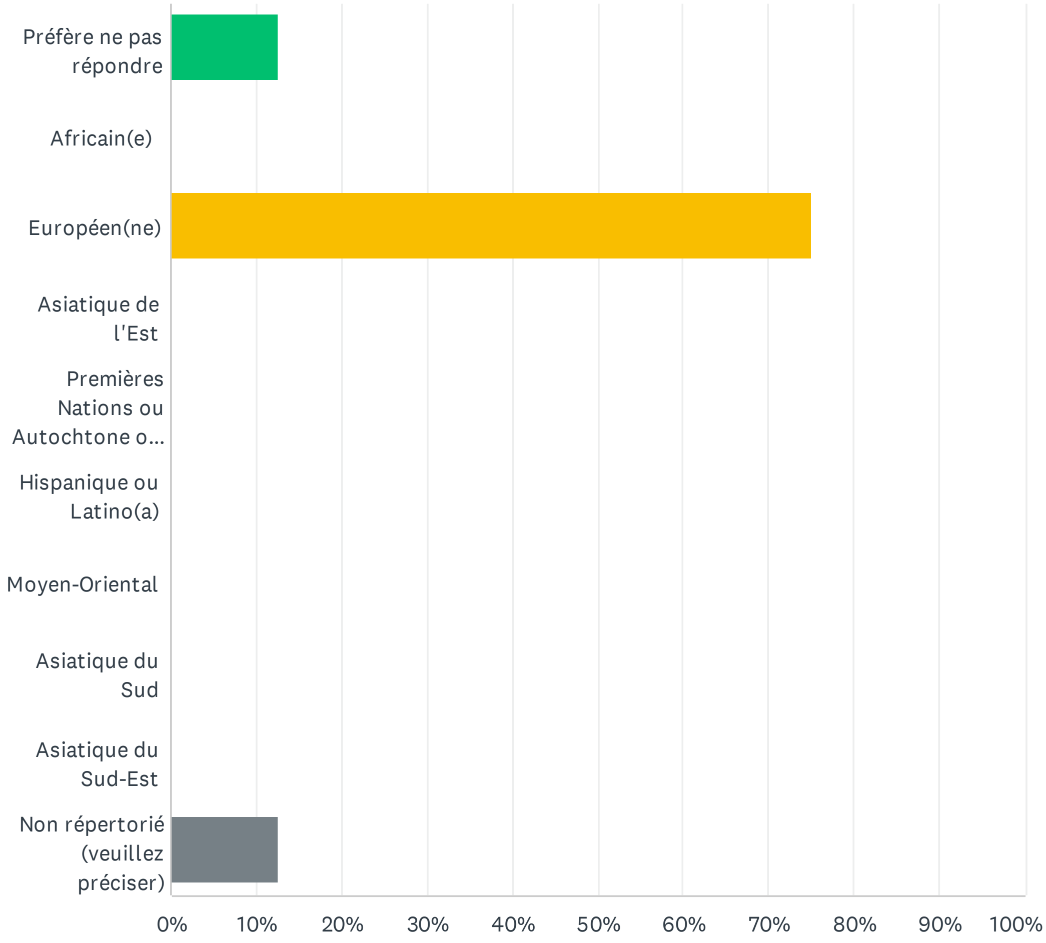

ANSWER CHOICES	RESPONSES	
Préfère ne pas répondre	12.50%	1
Africain(e)	0.00%	0
Européen(ne)	75.00%	6
Asiatique de l'Est	0.00%	0
Premières Nations ou Autochtone ou Métis	0.00%	0
Hispanique ou Latino(a)	0.00%	0
Moyen-Oriental	0.00%	0
Asiatique du Sud	0.00%	0
Asiatique du Sud-Est	0.00%	0
Non répertorié (veuillez préciser)	12.50%	1
Total Respondents: 8		

Q38 Veuillez préciser votre appartenance culturelle aux Premières Nations ou aux peuples autochtonesPlease specify your First Nations or Indigenous cultural background

Answered: 0   Skipped: 14

Q39 Avez-vous d'autres commentaires que vous aimeriez partager concernant votre expérience avec les compressions thoraciques guidées par l'ETO avant de passer à la prochaine partie du sondage?Do you have any additional comments you would like to share regarding your experience performing TEE-guided chest compressions before we move to the next part of the survey?

Answered: 2 Skipped: 12

Q40 Si vous souhaitez participer à une entrevue de suivi en français ou en anglais, veuillez fournir votre nom et votre adresse courriel :If you are interested in participating in a follow-up interview either in French or English, please provide you name and email address:

Answered: 0 Skipped: 14

ANSWER CHOICES	RESPONSES	
Nom	0.00%	0
Company	0.00%	0
Address	0.00%	0
Address 2	0.00%	0
City/Town	0.00%	0
State/Province	0.00%	0
ZIP/Postal Code	0.00%	0
Country	0.00%	0
Adresse courriel	0.00%	0
Phone Number	0.00%	0
